# Machine Learning Models With Prognostic Implications for Predicting Gastrointestinal Bleeding After Coronary Artery Bypass Grafting and Guiding Personalized Medicine: Multicenter Cohort Study

**DOI:** 10.2196/68509

**Published:** 2025-03-06

**Authors:** Jiale Dong, Zhechuan Jin, Chengxiang Li, Jian Yang, Yi Jiang, Zeqian Li, Cheng Chen, Bo Zhang, Zhaofei Ye, Yang Hu, Jianguo Ma, Ping Li, Yulin Li, Dongjin Wang, Zhili Ji

**Affiliations:** 1 Beijing Institute of Heart, Lung and Blood Vessel Diseases Beijing Anzhen Hospital Capital Medical University Beijing China; 2 Department of Acute Abdomen Surgery Beijing ChaoYang Hospital Capital Medical University Beijing China; 3 Department of General Surgery Beijing Chaoyang Hospital Capital Medical University Beijing China; 4 Department of Hepatobiliary and Pancreaticosplenic Surgery Beijing Chaoyang Hospital Capital Medical University Beijing China; 5 Department of Cardiovascular Surgery Nanjing Drum Tower Hospital Chinese Academy of Medical Science & Peking Union Medical College Nanjing China; 6 Department of Cardiovascular Surgery Nanjing Drum Tower Hospital The Affiliated Hospital of Nanjing University Medical School Beijing China; 7 Department of General Surgery Beijing Luhe Hospital Capital Medical University Beijing China; 8 Department of Cardiovascular Surgery Beijing Anzhen Hospital Capital Medical University Beijing China; 9 School of Instrumentation and Optoelectronic Engineering Beihang University Beijing China

**Keywords:** machine learning, personalized medicine, coronary artery bypass grafting, adverse outcome, gastrointestinal bleeding

## Abstract

**Background:**

Gastrointestinal bleeding is a serious adverse event of coronary artery bypass grafting and lacks tailored risk assessment tools for personalized prevention.

**Objective:**

This study aims to develop and validate predictive models to assess the risk of gastrointestinal bleeding after coronary artery bypass grafting (GIBCG) and to guide personalized prevention.

**Methods:**

Participants were recruited from 4 medical centers, including a prospective cohort and the Medical Information Mart for Intensive Care IV (MIMIC-IV) database. From an initial cohort of 18,938 patients, 16,440 were included in the final analysis after applying the exclusion criteria. Thirty combinations of machine learning algorithms were compared, and the optimal model was selected based on integrated performance metrics, including the area under the receiver operating characteristic curve (AUROC) and the Brier score. This model was then developed into a web-based risk prediction calculator. The Shapley Additive Explanations method was used to provide both global and local explanations for the predictions.

**Results:**

The model was developed using data from 3 centers and a prospective cohort (n=13,399) and validated on the Drum Tower cohort (n=2745) and the MIMIC cohort (n=296). The optimal model, based on 15 easily accessible admission features, demonstrated an AUROC of 0.8482 (95% CI 0.8328-0.8618) in the derivation cohort. In external validation, the AUROC was 0.8513 (95% CI 0.8221-0.8782) for the Drum Tower cohort and 0.7811 (95% CI 0.7275-0.8343) for the MIMIC cohort. The analysis indicated that high-risk patients identified by the model had a significantly increased mortality risk (odds ratio 2.98, 95% CI 1.784-4.978; *P*<.001). For these high-risk populations, preoperative use of proton pump inhibitors was an independent protective factor against the occurrence of GIBCG. By contrast, dual antiplatelet therapy and oral anticoagulants were identified as independent risk factors. However, in low-risk populations, the use of proton pump inhibitors (χ^2^_1_=0.13, *P*=.72), dual antiplatelet therapy (χ^2^_1_=0.38, *P*=.54), and oral anticoagulants (χ^2^_1_=0.15, *P*=.69) were not significantly associated with the occurrence of GIBCG.

**Conclusions:**

Our machine learning model accurately identified patients at high risk of GIBCG, who had a poor prognosis. This approach can aid in early risk stratification and personalized prevention.

**Trial Registration:**

Chinese Clinical Registry Center ChiCTR2400086050; http://www.chictr.org.cn/showproj.html?proj=226129

## Introduction

Approximately 1.5 million people worldwide undergo cardiac surgery each year, with coronary artery bypass grafting (CABG) being the most common procedure [1,2]. The incidence of gastrointestinal bleeding (GIB) after CABG (GIBCG) ranges from approximately 0.39% to 5.5% [3-8], increasing between 5% and >10% in patients treated with dual antiplatelet agents [9]. In the study by Yang et al [10], the observed incidence rate reached 22.4%. However, once GIBCG occurs, the mortality rate rises significantly, ranging from 8.8% to 38.0% [3-8], which is notably higher than that of surgical patients without GIB.

GIBCG has delayed and insidious characteristics that make early recognition difficult [4-6]. These patients often experience adverse events such as respiratory or renal failure and cardiac insufficiency, which can mask its clinical signs. Postoperative sedation may also obscure typical abdominal symptoms [6]. Following a GIBCG episode, continued administration of antiplatelet agents can exacerbate bleeding, whereas discontinuing antiplatelet therapy is associated with a higher risk of cardiovascular events and increased mortality rates [11]. Therefore, preventing and diagnosing GIBCG early are essential in clinical practice.

The PRECISE-DAPT (Predicting bleeding complications in patients undergoing stent implantation and subsequent dual antiplatelet therapy) score is one of the most commonly used clinical tools for predicting bleeding risk in patients with acute coronary syndrome. It assesses the risk of bleeding within 12 months following percutaneous coronary intervention [12]. Unlike percutaneous coronary intervention, CABG may cause GIB through different mechanisms, such as the effects of extracorporeal circulation and stress [5,13]. However, the applicability of these scores in patients undergoing CABG remains unclear.

Machine learning (ML), a subset of artificial intelligence, effectively identifies nonlinear relationships, deciphers intricate interactions, and manages multicollinearity among predictor variables [14-16]. ML has been widely applied in medicine, leveraging large volumes of patient data to build risk models. These models can predict disease onset, assess condition severity, and evaluate disease prognosis [17-19]. ML outperforms traditional scores in predicting GIB risk after antithrombotic therapy [20]. However, GIB risk prediction after cardiac surgery remains understudied. To date, only 1 study—a single-center, small-sample, traditional risk prediction model using intraoperative and postoperative clinical features—has reported on GIBCG risk prediction, but it does not allow for preoperative risk assessment [10]. In this study, we aimed to develop and evaluate ML-driven risk prediction models using preoperative clinical features to identify individuals at a high risk of GIBCG, thereby enabling proactive preventive measures.

## Methods

### Ethical Considerations

The Medical Ethics Committee of Beijing Anzhen Hospital, affiliated with Capital Medical University, approved the study protocol (approval number KS2023020). This study is retrospectively registered in the Chinese Clinical Trial Registry (ChiCTR2400086050), which includes GIB as the primary outcome along with other gastrointestinal complications. GIB was the main focus of this study due to its high morbidity and lethality. Informed consent was obtained from all participants. DJ obtained access to and download permission for the Medical Information Mart for Intensive Care IV (MIMIC-IV) database (ID: 59888302). All data have undergone deidentification processing to ensure anonymity.

### Patients and Study Design

The study included patients from 4 hospitals in China, with the inclusion criteria being CABG during hospitalization, age over 18 years, and availability of complete key information. Data were also obtained from the American Critical Care database, the MIMIC-IV, which met the same inclusion criteria [21]. Patients hospitalized at Beijing Anzhen Hospital between January 2018 and May 2023, Beijing Luhe Hospital between July 2019 and May 2023, and Beijing Chaoyang Hospital between May 2022 and April 2024 were included in the derivation cohort. Additionally, 3466 patients prospectively recruited at Beijing Anzhen Hospital between May 2023 and April 2024 were included as a prospective cohort within the same derivation cohort. Patients hospitalized at Nanjing Drum Tower Hospital between October 2010 and May 2023, along with data from MIMIC-IV, were included in the external validation cohort. The exclusion criteria were as follows: patients diagnosed preoperatively with GIB and those whose bleeding cause could not be identified due to comorbidities such as severe liver disease or gastrointestinal malignancy. Figure S1 in Multimedia Appendix 1 details the patient selection process for the derivation and external validation cohorts.

### Sample Size Calculation

When developing prediction models, a common practice for determining the required sample size is to ensure at least 10 events per candidate predictor parameter. Additionally, we followed the 4-step procedure proposed by Riley et al [22] to calculate the required sample size. The calculation process, formulas, and results are presented in Table S1 in Multimedia Appendix 1.

### Definition of Outcome

The primary outcome was the occurrence of GIB after CABG, defined as (1) hematemesis (including bright red blood or coffee-ground emesis), melena, hematochezia, or positive occult blood tests in either gastric fluid or stool; and (2) bleeding foci identified during endoscopic examination [23-25]. The secondary outcome was the in-hospital mortality rate.

### Predictive Features

Admission variables were used as predictive features. Table S2 in Multimedia Appendix 1 provides the definitions of each feature. Data extraction for patients undergoing CABG and the diagnosis of comorbidities were based on International Classification of Diseases 9th/10th Revision (ICD-9/10) disease codes (Table S3 in Multimedia Appendix 1).

### Statistical Analysis

Data analysis was performed using SPSS Statistical Software version 26.0(IBM Corp.). Normally distributed continuous variables are presented as means with SDs, while skewed continuous variables are presented as medians with IQRs. For statistical analysis, the independent samples *t* test was used to compare normally distributed continuous variables, and the Mann-Whitney *U* test was used for skewed distributions. Categorical variables are presented as numbers with percentages and were compared using the chi-square or Fisher exact test. Significant variables from the univariate analysis were selected for multivariate unconditional logistic regression. Differences were considered statistically significant at *P*<.05. Missing values were imputed using multiple imputation methods in R version 4.1.0 9 (R Foundation). Subsequent feature screening and model construction were performed using Python version 3.7.0 (Python Foundation). Table S4 in Multimedia Appendix 1 presents the data types and missing values for each feature.

### Feature Selection

**Figure 1:**
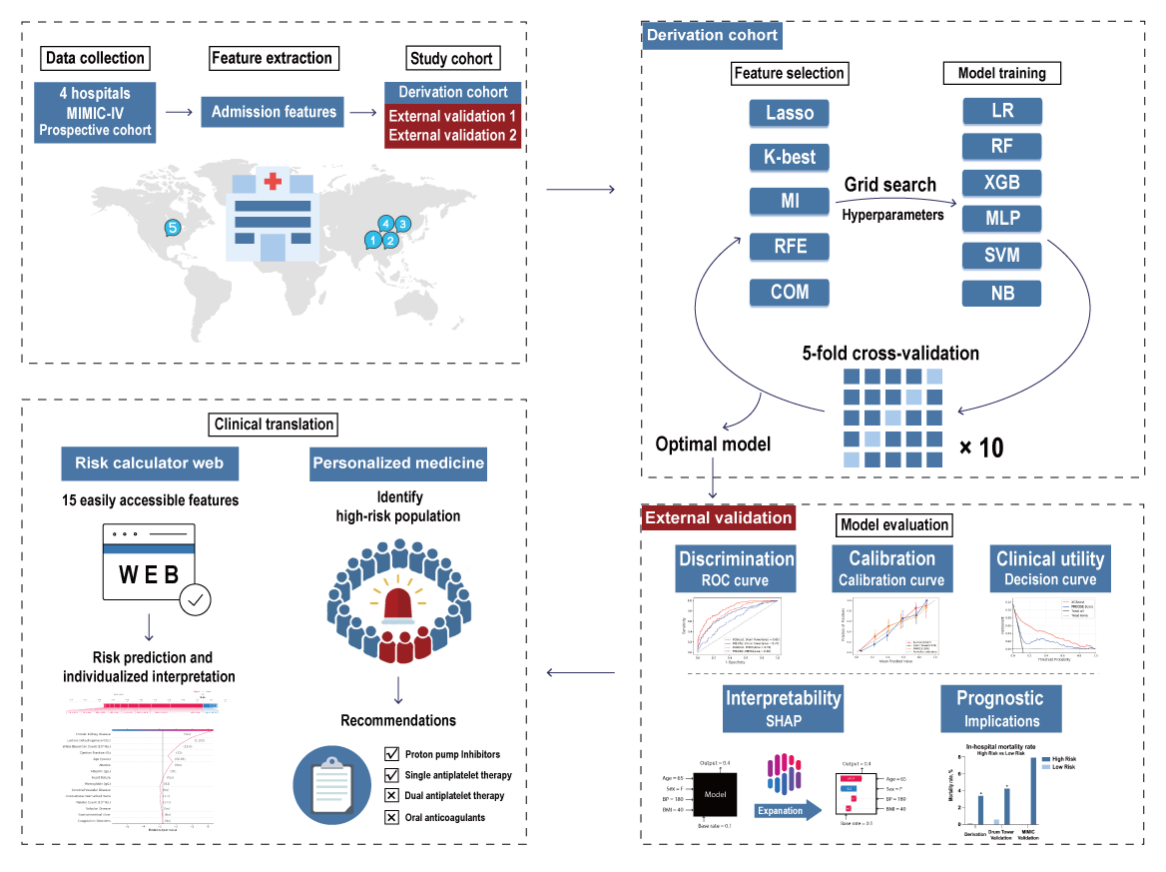
Flowchart of the study. The study process is summarized as follows: data were collected from multiple centers, patient admission features were extracted, and the study cohort was defined. A derivation cohort was formed from 3 centers and a prospective cohort, which were used for feature screening and model construction. The optimal model was selected using multiple evaluation metrics and externally validated with the Drum Tower and MIMIC cohorts. Finally, the model was developed into a user-friendly web page to facilitate clinical use and guide treatment decisions. Abbreviations: COM: combined method, including features appearing more than three times in the first four feature selection methods; K-Best: k-best feature selection; LASSO: least absolute shrinkage and selection operator; LR: logistic regression; MI: mutual information; MIMIC IV: medical information mart for intensive care IV; MLP: multilayer perceptron; NB: naive Bayes; RF: random forest; RFE: recursive feature elimination; ROC curve: receiver operating characteristic curve; SHAP: Shapley additive explanations; SVM: support vector machine; XGB: XGBoost (extreme gradient boosting).

Figure 1 presents the complete study flowchart. We employed 4 distinct feature selection methods to identify candidate features for model development: least absolute shrinkage and selection operator (LASSO), k-best feature selection (K-Best), random forest recursive feature elimination (RFE), and the mutual information algorithm [26]. A fifth method was a combined approach that selected features appearing in at least three of the four feature selection methods.

### Model Development and Validation

Based on feature engineering, we applied 6 supervised ML algorithms: multilayer perceptron, naive Bayes, random forest, Extreme Gradient Boosting (XGBoost; also called XGB), logistic regression, and support vector machines. By combining 5 feature selection methods with these 6 classifiers, we built 30 models (5 × 6 = 30) and optimized hyperparameters for each model using a 5-fold grid search cross-validation on the derivation cohort. Grid search systematically explores all possible combinations of hyperparameters, while 5-fold cross-validation evaluates each combination’s performance across different validation subsets. The optimal hyperparameter combination for each model was selected based on the highest performance across cross-validation folds. The hyperparameter settings, ranges, and rationale for each algorithm are detailed in Table S5 in Multimedia Appendix 1. The final model selection was based on a comprehensive evaluation of multiple performance metrics, including the average and SD of both the area under the receiver operating characteristic curve (AUROC) and the Brier score [27]. AUROC measures the model’s ability to distinguish between different classes, which is particularly important in imbalanced data sets. A higher AUROC value indicates better class discrimination. The Brier score, by contrast, assesses both the accuracy and calibration of predicted probabilities by quantifying the difference between predicted probabilities and actual outcomes. Using these metrics provides a well-rounded evaluation of model performance in terms of both discriminative power and predictive accuracy. The average and SD of AUROC and Brier score were calculated based on all validation results from 5-fold, 10-time cross-validation. The ideal model performance is characterized by an average AUROC close to 1, a Brier score close to 0, and low SDs for both metrics. To quantify overall model performance, we first normalized the average AUROC. Next, we normalized the average Brier score and the SDs of both AUROC and Brier score by subtracting them from 1. The comprehensive model score was then obtained by averaging these 4 values. Based on this score, we selected the optimal combination of the feature selection method and ML model for further evaluation in external validation.

### Model Evaluation

In the external validation cohort, we further assessed the performance of the final prediction models using AUROC, calibration plots, and decision curve analysis (DCA). Model discrimination was evaluated using AUROC, and we compared the discriminative performance of the optimal ML model with PRECISE-DAPT scores. Calibration was demonstrated through calibration plots and quantified using the Brier score, while clinical utility was assessed using DCA [28]. We categorized bleeding severity, defining severe GIB as the presence of marked symptoms such as hematemesis, melena, or hematochezia, or the identification of bleeding foci during endoscopic examination. Patients with positive occult blood tests in gastric fluid or stool were classified as having mild bleeding. Additionally, we assessed the model’s ability to predict severe bleeding within each study cohort.

### Model Interpretations

ML modeling often operates in a “black box” environment, where the complexity and multidimensionality of algorithms make it difficult to clearly elucidate the internal mechanisms driving accurate predictions for specific patient groups. To enhance model interpretability, we used the Shapley Additive Explanations (SHAP) algorithm [29,30]. SHAP is a game theory-based model explanation method that provides consistent and locally precise attribution values—known as SHAP values—for each feature in the model. This approach highlights the significance of individual features and explains the model’s decision-making process. Higher SHAP values indicate a greater likelihood of GIBCG.

### Clinical Application

We determined the model’s optimal cutoff values by identifying the maximum Youden index (sensitivity + specificity – 1) in the derivation cohort. Using these cutoff values, patients were classified into high- and low-risk groups. Subgroup analyses were conducted based on preoperative medication use across different risk groups. Finally, we developed a web-based interface integrating the optimal ML model, which utilizes questionnaire-guided responses for risk assessment.

## Results

### Patient Characteristics

The calculated sample size was 5667. However, to meet the requirements of ML training, we used a data set that far exceeded this minimum to ensure optimal model performance. In total, we included 16,440 patients, with 13,399 in the derivation cohort and 3041 in the external validation cohort. Baseline characteristics for these groups are presented in Table 1. In the derivation cohort (N=13,399), 803 patients (5.99%) developed GIBCG. The external validation cohort comprised data from Nanjing Drum Tower Hospital and MIMIC, incorporating both Chinese and American centers. The Drum Tower data set (n=2745) was designated as external validation cohort 1, in which 179 patients (6.52%) developed GIBCG. The MIMIC data set (n=296) was designated as external validation cohort 2, with 176 patients (59.5%) developing GIBCG. Baseline characteristics stratified by GIBCG occurrence are compared in Tables S6-S8 in Multimedia Appendix 1.

**Table 1:** Baseline characteristics of the derivation and external validation cohorts.

Demographic variables				Derivation cohort		External validation cohort		
				Multicenter (n=13,399)		Drum Tower (n=2745)		MIMIC^a^ (n=296)
Age, median (IQR), years				63 (11)		70 (14)		74 (15)
**Sex, n (%)**								
	Male			10,108 (75.44)		1921 (69.98)		198 (66.89)
	Female			3291 (24.56)		824 (30.02)		98 (33.11)
BMI, median (IQR)				25.59 (4.09)		24.4 (4.3)		30.09 (7.31)
Gastrointestinal bleeding history, n (%)				102 (0.76)		29 (1.06)		30 (10.14)
After percutaneous coronary intervention, n (%)				1578 (11.78)		262 (9.54)		28 (9.46)
**Chronic comorbidities, n (%)**								
	Anemia			1704 (12.72)		319 (11.62)		122 (41.22)
	Coagulation disorders			147 (1.10)		17 (0.62)		92 (31.08)
	Hypertension			6238 (46.56)		1691 (61.60)		131 (44.26)
	Atrial fibrillation			618 (4.61)		258 (9.40)		185 (62.50)
	Diabetes			5314 (39.66)		832 (30.31)		100 (33.78)
	Heart failure			5114 (38.17)		1114 (40.58)		146 (49.32)
	Cerebral vascular disease			2045 (15.26)		484 (17.63)		53 (17.91)
	Peripheral vascular disease			347 (2.59)		196 (7.14)		71 (23.99)
	Chronic kidney disease			614 (4.58)		133 (4.85)		113 (38.18)
	Gastrointestinal ulcer			209 (1.56)		37 (1.35)		11 (3.72)
	Gastritis			155 (1.16)		85 (3.10)		10 (3.38)
	Hyperlipidemia			8413 (62.79)		1689 (61.53)		201 (67.91)
	Valvular disease			2087 (15.58)		433 (15.77)		107 (36.15)
**Admission examination, median (IQR)**								
	White blood cell count (×10^9^/L)			6.89 (2.83)		6.3 (2.4)		11.35 (7.68)
	Red blood cell count (×10^12^/L)			4.41 (0.77)		4.38 (0.71)		3.25 (0.79)
	Platelet count (×10^9^/L)			207 (79)		191 (73.5)		176.5 (136)
	Hemoglobin (g/L)			136 (25)		134 (22)		98 (23)
	Hematocrit (proportion of 1.0)			0.396 (0.069)		0.398 (0.06)		0.295 (0.063)
	Alanine aminotransferase (µkat/L)			0.33 (0.3)		0.36 (0.32)		0.4 (0.56)
	Aspartate aminotransferase (µkat/L)			0.32 (0.17)		0.37 (0.24)		0.62 (0.99)
	Bilirubin total (µmol/L)			11.01 (6.4)		10.8 (6.9)		10.26 (13.25)
	Albumin (g/L)			42.7 (4.8)		39.6 (4)		33.07 (6.09)
	Urea (mmol/L)			5.77 (2.28)		6.1 (2.5)		9.64 (8.93)
	Creatinine (μmol/L)			75 (21.7)		71 (25.55)		106.08 (79.56)
	Prothrombin time (seconds)			11.4 (1.1)		11.5 (1.3)		14.4 (4.58)
	Activated partial thromboplastin time (seconds)			31 (4.4)		27.8 (4.1)		31.8 (12.8)
	International normalized ratio			1.01 (0.09)		1 (0.12)		1.3 (0.5)
	Lactate dehydrogenase (µkat/L)			2.92 (0.77)		3.34 (1.45)		5.06 (3.01)
**Admission examination**								
	**Ejection fraction, n (%)**							
		≥55%	10,611 (79.19)		1572 (57.27)		109 (36.82)	
		45%-55%	1772 (13.22)		579 (21.09)		76 (25.68)	
		30%-44%	968 (7.22)		543 (19.78)		72 (24.32)	
		<30%	48 (0.36)		51 (1.86)		39 (13.18)	
PRECISE-DAPT^b^ score, median (IQR)				12.3 (11.36)		14.53 (10.78)		40.48 (22.46)
In-hospital mortality, n (%)				123 (0.92)		69 (2.51)		22 (7.43)

^a^MIMIC: Medical Information Mart for Intensive Care.

^b^PRECISE-DAPT: predicting bleeding complications in patients undergoing stent implantation and subsequent dual antiplatelet therapy.

### Predictive Features

Five feature selection methods—LASSO, K-Best, RFE, mutual information algorithm, and a combined approach—were used to optimize the feature set (Table S9 in Multimedia Appendix 1). The features identified through these methods were incorporated as predictors of GIBCG and subsequently used in the ML model for further evaluation.

### Model Performance

**Figure 2:**
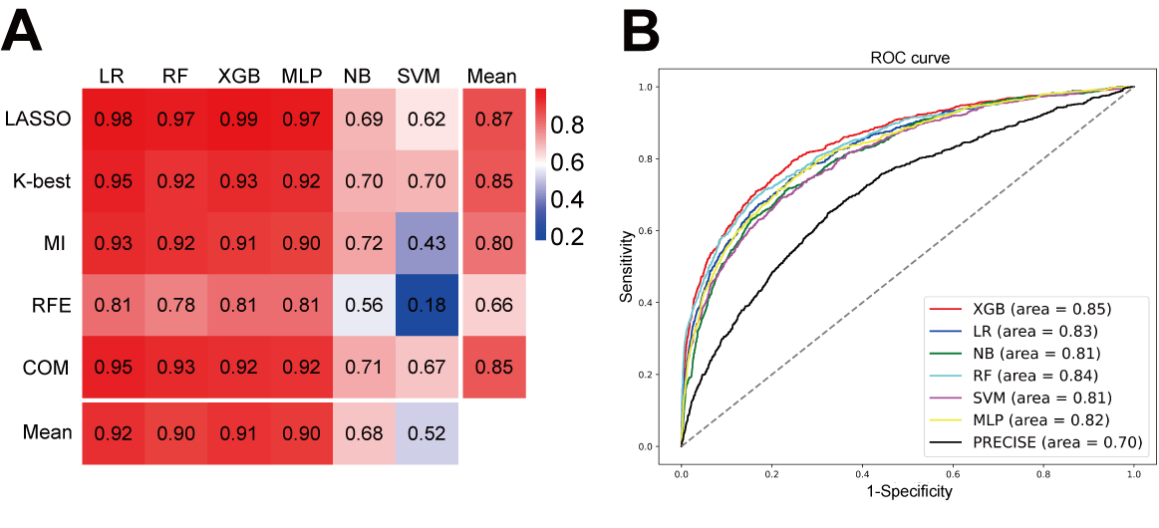
Performance of machine learning models. (A) Heatmaps illustrating the predictive performance (model score) of different combinations of feature selection methods (rows) and classification algorithms (columns). (B) Receiver operating characteristic curves of various models following LASSO feature selection. Abbreviations: COM: combined method; K-Best: k-best feature selection; LASSO: least absolute shrinkage and selection operator; LR: logistic regression; MI: mutual information; MLP: multilayer perceptron; NB: naive Bayes; PRECISE: “predicting bleeding complications in patients undergoing stent implantation and subsequent dual antiplatelet therapy” score; RF: random forest; RFE: recursive feature elimination; SVM: support vector machine; XGB: XGBoost (extreme gradient boosting).

Using grid search and cross-validation, we identified the optimal hyperparameters for each model configuration (Table S10 in Multimedia Appendix 1). Table S11 presents the average AUROC and its 95% CI for each model in the training set, constructed from 4 folds of the 5-fold cross-validation in the derivation cohort. Table S12 displays the AUROC for the internal validation set, which consists of the remaining fold. Similarly, Tables S13 and S14 report the mean Brier score and its 95% CI for the training and internal validation sets, respectively. We constructed 6 models for each of the 5 feature selection methods, yielding 30 (5×6) models. Figure 2A presents a heatmap of comprehensive scores, ranging from 0.1789 to 0.9938. Vertically, models developed using the LASSO method achieved an average score of 0.8709, outperforming those built with other feature selection techniques. Horizontally, XGBoost demonstrated superior performance across feature selection methods, with an average score of 0.9128. Notably, the LASSO-XGBoost combination attained the highest score of 0.9938, making it the top-performing model. Figure 2B illustrates the AUROC of models following LASSO feature selection, with XGBoost achieving the highest AUROC of 0.8482 (95% CI 0.8328-0.8618, bootstrap), outperforming all other models.

### Model Evaluation

**Figure 3:**
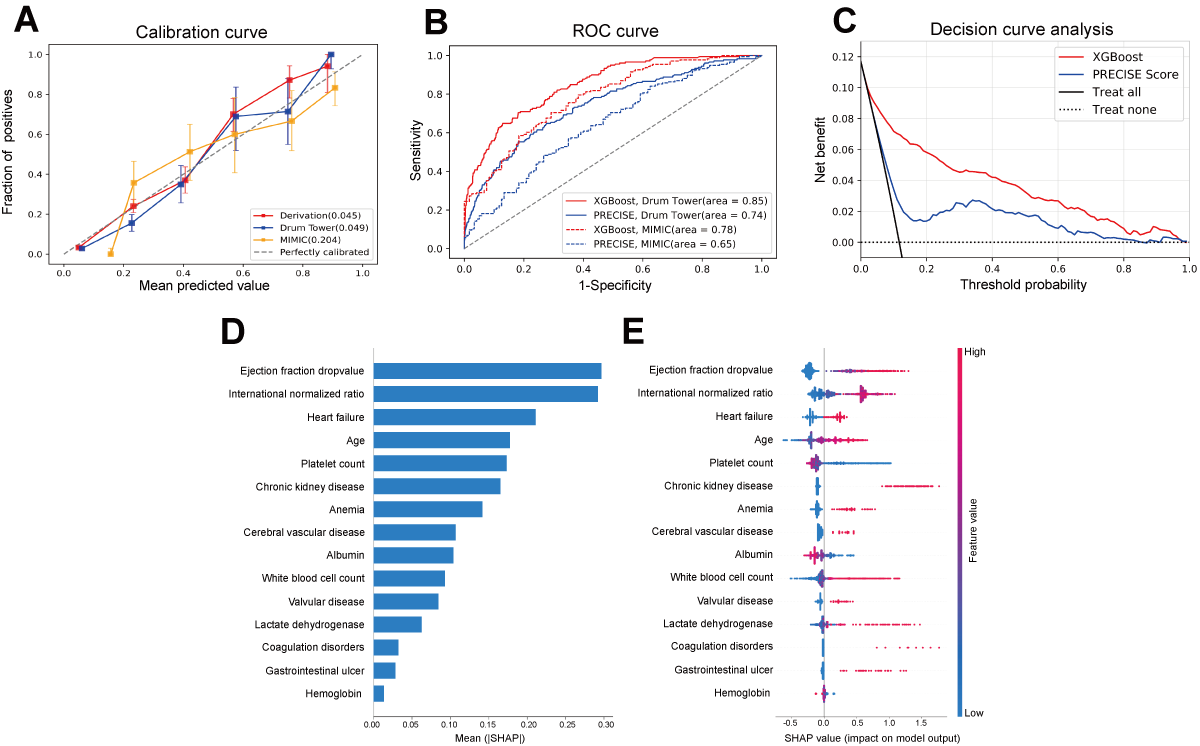
Evaluation of the optimal model and its interpretability. (A) Calibration plots of XGBoost (Extreme Gradient Boosting) in the derivation cohort and 2 independent external validation cohorts. The Brier score is reported in the lower-right legend (a smaller value indicates better calibration). (B) Comparison of the area under the receiver operating characteristic curve (AUROC) for XGBoost and the PRECISE-DAPT (predicting bleeding complications in patients undergoing stent implantation and subsequent dual antiplatelet therapy) score in the Drum Tower and MIMIC (Medical Information Mart for Intensive Care) validation cohorts. (C) Comparison of clinical decision curves for XGBoost and the PRECISE-DAPT score in the combined external validation cohort. (D) Bar chart of the mean absolute Shapley Additive Explanations (SHAP) value for each predictor.(E) SHAP summary plot. The SHAP value along the x axis serves as a standardized measure of feature influence within the model. Each row of critical features illustrates the contributions of all patients to the outcome, with dots in different colors: red representing high-risk values and blue representing low-risk values.

The final prediction model was evaluated using 2 independent external cohorts from China and the United States. Figure 3A presents calibration plots for the XGBoost model across the derivation cohort and both external validation cohorts. Given the higher positive rate in the MIMIC cohort, we applied Platt scaling to calibrate predicted probabilities for this data set. In the derivation cohort, the XGBoost model achieved a Brier score of 0.0446 (95% CI 0.0419-0.0474). The Brier score was 0.0489 (95% CI 0.0431-0.0549) in the Drum Tower validation cohort and 0.2044 (95% CI 0.1787-0.2322) in the MIMIC validation cohort. These results indicate that our model demonstrates the highest calibration in the 2 Chinese cohorts while maintaining satisfactory calibration in the American cohort. Figure 3B compares the AUROC of the XGBoost model and the PRECISE-DAPT score within the Drum Tower validation cohort. The AUROC for XGBoost was 0.8513 (95% CI 0.8221-0.8782), exceeding that of the PRECISE-DAPT score (0.7427, 95% CI 0.7028-0.7836). Similarly, in the MIMIC validation cohort (dashed curves in Figure 3B), XGBoost achieved an AUROC of 0.7811 (95% CI 0.7275-0.8343), outperforming the PRECISE-DAPT score (0.6460, 95% CI 0.5863-0.7123). Figure 3C presents a DCA integrating both external validation cohorts, showing that the net benefit of XGBoost surpasses that of the PRECISE-DAPT score.

The proportion of patients with severe bleeding among those with GIBCG is presented for each study cohort (Figure S2 in Multimedia Appendix 1). The model demonstrated excellent discriminative ability for severe GIB, with AUROCs of 0.8912 (95% CI 0.8626-0.9196) in the derivation cohort, 0.8817 (95% CI 0.8249-0.9296) in the Drum Tower validation cohort, and 0.8388 (95% CI 0.7749-0.8931) in the MIMIC validation cohort (Figure S3 in Multimedia Appendix 1).

The MIMIC cohort consists of critically ill patients; therefore, we assessed disease severity using intensive care unit stay duration, Sequential Organ Failure Assessment (SOFA) score on the first day, and the Charlson Comorbidity Index for further subgroup analysis (Figure S4 in Multimedia Appendix 1). In the figure, the red line represents the intensive care unit stay duration cutoff, with the model’s AUROC calculated for patients below this threshold. The blue and green lines indicate the SOFA score and the Charlson Comorbidity Index, respectively. Line charts illustrate AUROC changes across different cutoff values for these 3 indicators. The results show that as disease severity increases (ie, with higher cutoff values), the AUROC gradually decreases, suggesting that the model performs better in predicting less severe cases than more severe ones.

### Model Interpretability Using SHAP Values

Figure 3D presents a bar chart displaying the average absolute SHAP values for each predictive feature, with higher SHAP values indicating greater feature influence. Figure 3E provides a SHAP summary plot illustrating how each predictive feature impacts the XGBoost model’s predictions. A decrease in ejection fraction, international normalized ratio (INR), platelet count, albumin, and hemoglobin levels, and the presence of heart failure, advanced age, chronic kidney disease, anemia, cerebrovascular disease, valvular disease, elevated white blood cell count, increased lactate dehydrogenase levels, coagulation disorders, and gastrointestinal ulcer were associated with a higher predicted probability of GIBCG.

### Prognostic Implications

We evaluated the prognostic implications across all 16,440 hospitalized patients from the derivation and external validation cohorts. In the derivation cohort, an optimal cutoff value of 0.0592 was identified based on the maximum Youden index to differentiate between high- and low-risk populations, and the same threshold was applied to the external validation cohort. Univariate and multivariate analyses of risk factors for mortality were conducted across all cohorts (Table S15 in Multimedia Appendix 1). The findings indicate that the model-identified risk levels (high risk vs low risk) serve as an independent risk factor for in-hospital mortality (odds ratio [OR] 2.98, 95% CI 1.784-4.978; *P*<.001). The model’s risk probability output was also an independent risk factor for in-hospital mortality (OR 1.017, 95% CI 1.004-1.031; *P*=.009). Compared with the low-risk population, the high-risk population exhibited significantly higher mortality rates in the derivation cohort (106/4534, 2.34% versus 17/8865, 0.19%; *χ*^2^_1_=151.91, *P*<.001), Drum Tower validation cohort (60/1354, 4.43% versus 9/1391, 0.65%; *χ*^2^_1_=40.09, *P*<.001), and MIMIC validation cohort (22/278, 7.91% versus 0/18, 0%; *χ*^2^_1_=0.60, *P*=.44; Figure S5 in Multimedia Appendix 1). Although the PRECISE-DAPT score is an independent risk factor for in-hospital mortality (Table S15 in Multimedia Appendix 1), Figure S6 in Multimedia Appendix 1 presents the receiver operating characteristic curves for predicting in-hospital mortality using both our model and the PRECISE-DAPT score. The AUROC for the risk probability predicted by our model was 0.8642 (95% CI 0.8384-0.8860), compared with 0.7591 (95% CI 0.7266-0.7939) for the PRECISE-DAPT score, demonstrating the superior prognostic performance of our model.

### Guiding Personalized Medicine

Given the substantial variations in prognosis across different risk populations, we conducted a subgroup analysis comparing preoperative medications between CABG patients with and without GIB to explore potential intervention strategies. In all cohorts, preoperative use of proton pump inhibitors (PPIs; *χ*^2^_1_=53.45, *P*<.001), dual antiplatelet therapy (DAPT; *χ*^2^_1_=16.53, *P*<.001), and oral anticoagulants (OACs; *χ*^2^_1_=29.87, *P*<.001) was significantly associated with the occurrence of GIBCG. However, in the low-risk population, PPIs (*χ*^2^_1_=0.13, *P*=.72), DAPT (*χ*^2^_1_=0.38, *P*=.54), and OACs (*χ*^2^_1_=0.15, *P*=.69) were not significantly associated (Table S16 in Multimedia Appendix 1). In the high-risk population, univariate analysis identified preoperative use of PPIs and single antiplatelet therapy (SAPT) as protective factors against GIBCG, whereas DAPT and OACs were associated with an increased risk. Furthermore, multivariate analysis confirmed that PPIs, DAPT, and OACs were independent correlates of GIBCG occurrence (Table S17 in Multimedia Appendix 1)

These findings were further validated in the Drum Tower and MIMIC validation cohorts (Table S16 in Multimedia Appendix 1), confirming the universality and robustness of our cutoff value. The analysis of GIBCG risk factors in the Drum Tower validation cohort aligned with those in the derivation cohort, particularly regarding preoperative medication (Table S18 in Multimedia Appendix 1). Although the MIMIC validation cohort, derived from the Critical Care Database, differed from the other 2 cohorts, its analysis yielded largely consistent results. Specifically, preoperative use of PPIs (OR 0.517, 95% CI 0.281-0.95; *P*=.03), SAPT (OR 0.56, 95% CI 0.342-0.915; *P*=.02), and DAPT (OR 2.821, 95% CI 1.12-7.106; *P*=.03) was significantly associated with GIBCG occurrence in the high-risk population (Table S19 in Multimedia Appendix 1).

### Clinical Application

**Figure 4:**
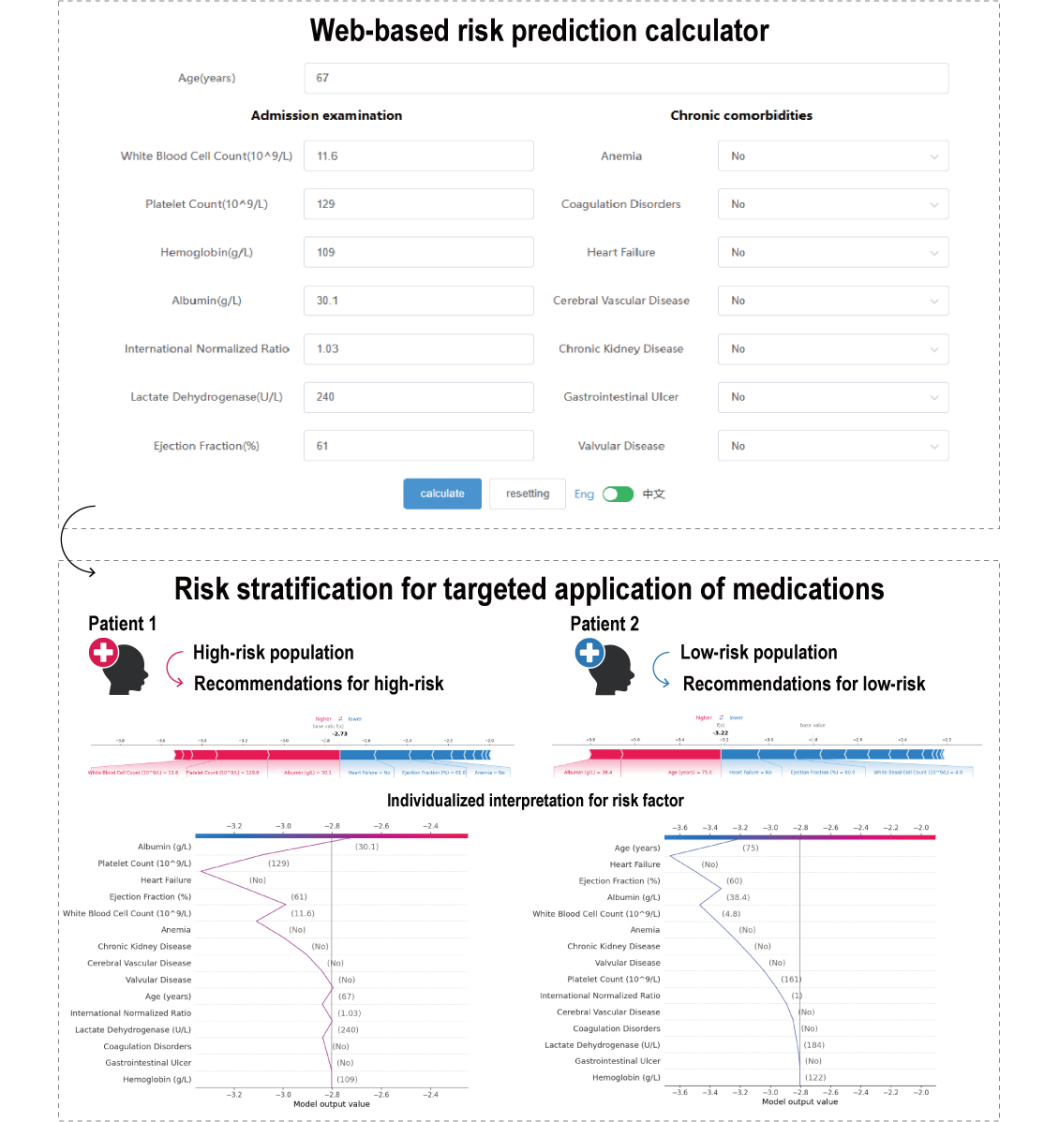
Web-based calculator of the XGBoost (Extreme Gradient Boosting) model for risk stratification and personalized medicine. The web-based risk calculator predicts risk accurately online and in real time, providing a convenient tool for clinical doctors. It utilizes 15 features, including patient age, comorbidities, and initial examination results upon admission, to predict the risk of gastrointestinal bleeding in cardiac surgery patients. Additionally, it provides individualized interpretations of risk factors, outputs the patient's risk level, facilitates risk stratification, and guides targeted medication use.

The XGBoost model was ultimately selected as the algorithm for the web-based calculator available on our website (Figure 4; also see [31]). Users input 15 admission features into the questionnaire interface, and the web page automatically predicts the patient’s GIBCG risk level. The results section displays the predicted risk along with its classification. Simultaneously, the calculator generates a force plot to interpret the prediction, highlighting the features influencing the decision. SHAP values provide insights into the contribution of each feature to the predicted risk of GIBCG: blue features on the right drive the prediction toward “non-GIB,” while red features on the left indicate a higher likelihood of “GIB.” For example, when we entered the admission features of 2 patients from the Drum Tower cohort, the calculator provided their risk predictions along with corresponding interpretation plots (Figure 4). At the time of admission, patient 1 presented with decreased albumin, elevated white blood cell counts, and a low platelet count within the normal range. These 3 factors were identified as the primary contributors to the increased GIBCG risk and warrant special clinical attention. The model classified patient 1 as high risk and recommended a preventive strategy. Preoperative PPIs use may be considered, and for patients on DAPT or OACs, an early transition to SAPT or alternative therapies is advised. By contrast, although patient 2 is older, most of their predictive factors were within normal ranges, leading to a classification as low risk. For patients with indications, antiplatelet therapy can be continued until preoperative cessation to mitigate the risk of adverse cardiovascular events. This personalized medical approach may help reduce GIBCG risk, improve patient prognosis, and enhance the overall quality of health care services.

## Discussion

### Principal Findings

In this multicenter study, we developed and validated ML-based prediction models for GIBCG. After evaluating various feature selection methods and ML algorithms, we identified LASSO for feature selection and XGBoost for model development as the optimal combination. The final model, utilizing 15 admission features, demonstrated strong discriminative performance, calibration, and clinical utility across both the derivation and external validation cohorts. The top 5 predictive features for GIBCG were ejection fraction, INR, chronic heart failure, age, and platelet count, in that order. To our knowledge, this study is the first to develop a model that accurately predicts GIBCG using admission features. Furthermore, our model is prognostically relevant, as it identifies high-risk individuals with an increased postoperative mortality rate, underscoring the need for clinical vigilance. By enabling early risk stratification and targeted prevention, our ML model may help mitigate the risk of GIBCG.

In the derivation and Drum Tower validation cohorts, the prevalence of GIBCG was 803 out of 13,399 (5.99%) patients and 179 out of 2745 (6.52%) patients, respectively, slightly higher than previous epidemiological estimates. This discrepancy may stem from our inclusion of patients with positive occult blood tests as positive cases. While these patients have no overt bleeding, early identification and preventive measures may help avert the progression to significant bleeding. Including occult blood positivity in the outcome definition enhances the model’s sensitivity, enabling earlier risk detection and management. Furthermore, subgroup analysis demonstrated that the model performed better in identifying patients with more severe bleeding, reinforcing its utility for early screening of high-risk patients.

The model developed in the derivation cohort was validated in a large, independent external validation cohort (Drum Tower), consistently demonstrating excellent performance. In intensive care, stress-related GIB remains a significant concern [32,33]. To further assess the model’s generalization capability across different populations, we tested it on the MIMIC cohort. This introduced potential biases in the following aspects: (1) the incidence of GIBCG in the MIMIC cohort was higher than in the other 2 cohorts, likely due to the greater severity of illness among these patients and the absence of fecal occult blood test results in some cases, which may have led to selection bias; (2) the AUROC for predicting GIBCG in the MIMIC cohort was lower than in the other cohorts. This discrepancy is primarily due to the higher proportion of critically ill patients in the MIMIC cohort. Analysis reveals that the model performs better for the mild subgroup than for the severe cases (Figure S4 in Multimedia Appendix 1). Furthermore, we conducted a subgroup analysis focusing on severe GIB (Figure S3 in Multimedia Appendix 1). When the outcome was defined as severe GIB, the model’s AUROC in the MIMIC cohort improved substantially, reaching 0.8388. These findings demonstrate that the model has predictive value in critically ill patient populations, particularly in identifying patients at risk for severe GIB.

Currently, no dedicated tool exists for assessing GIB risk in CABG patients. To address this gap, we developed a new predictive model and compared it with the PRECISE-DAPT score, a commonly used tool in internal medicine for evaluating bleeding risk in patients receiving DAPT [12]. Across all study cohorts, our model demonstrated superior performance, as evidenced by its higher AUROC and greater net benefit, as shown in the receiver operating characteristic and DCA curves. Furthermore, our model has been validated as a predictor of patient prognosis. In addition, we compared its predictive performance for in-hospital mortality with that of the PRECISE-DAPT score, demonstrating a significantly higher AUROC for our model, underscoring its superior prognostic capability. The PRECISE-DAPT score, developed using Cox regression, is widely used to predict bleeding risk in DAPT patients. However, its reliance on clinical variables that exclude cardiovascular function and gastrointestinal status may limit its applicability in patients undergoing cardiac surgery or in predicting GIB risk. By contrast, ML excels at capturing nonlinear relationships and complex interactions between variables, enabling our model to focus specifically on predicting GIBCG with superior performance. However, our model has limitations. It requires validation in larger-scale prospective cohorts to further establish its reliability. Additionally, its interpretability remains a challenge, making it difficult for clinicians to fully understand the reasoning behind its predictions. To address this, we incorporated SHAP to enhance explainability, as detailed in the following sections.

High-risk patients with GIBCG identified by the model have a significantly increased risk of death and should receive heightened clinical attention. Early identification enables timely interventions, such as preventive medication and optimized perioperative management, to mitigate GIBCG risk and improve prognosis. Strategies include PPIs use, risk factor management, and *Helicobacter pylori* eradication, which have been shown to reduce antithrombotic therapy–associated GIB [34]. Current guidelines recommend prophylactic PPIs use in cardiac surgery patients to minimize gastrointestinal adverse events, with a class Ⅱa level of evidence [35].

Additionally, managing antithrombotic therapy in high-risk patients with GIBCG should be optimized. Aspirin and clopidogrel are the most commonly used antiplatelet agents, and patients experiencing acute GIB while on aspirin are generally advised to continue therapy whenever possible [36-38]. During the perioperative period of CABG, aspirin can typically be maintained, except in cases of extremely high bleeding risk [39,40]. For patients scheduled for elective CABG who are receiving P2Y12 receptor blockers (eg, clopidogrel, prasugrel, ticagrelor), guidelines recommend discontinuing these agents 5-7 days before surgery to reduce the bleeding risk [41,42]. In patients with an elevated bleeding risk, discontinuation of antiplatelet therapy may be considered even earlier [36,37,43]. Furthermore, the concomitant use of anticoagulants and antiplatelet agents should be avoided whenever possible due to the significantly increased risk of GIB [44]. Existing evidence suggests that prophylactic PPIs use may be beneficial for high-risk patients undergoing CABG. For those already receiving DAPT or OACs preoperatively, an earlier transition to SAPT or alternative therapies should be considered to mitigate bleeding risk.

Additionally, our findings reinforce this evidence, demonstrating that preoperative PPIs use independently protects high-risk patients from developing GIBCG, whereas DAPT and OACs independently increase the risk. The protective effect of PPIs is primarily attributed to their irreversible binding to proton pumps in the secretory tubules and vesicles of gastric parietal cells, leading to H^+^/K^+^-ATPase inhibition and subsequent suppression of gastric acid secretion [45]. This helps reduce gastric mucosal damage, prevent ulcer formation, and lower the risk of stress ulcer bleeding [46,47]. Moreover, the suppression of gastric acid secretion by PPIs significantly increases gastric pH, preventing the conversion of pepsinogen to pepsin, which helps maintain clot stability and promotes hemostasis [48,49]. By contrast, antiplatelet agents heighten the risk of GIBCG. Aspirin, for instance, disrupts the gastric mucosal protective layer and induces oxidative stress and apoptosis in epithelial cells, leading to direct gastrointestinal injury [50,51]. It also inhibits cyclooxygenase (COX)-1 and COX-2, reducing prostaglandin production via COX-1, which weakens mucosal protection and contributes to indirect gastrointestinal damage [52]. Additionally, aspirin may impact the gut microbiota, triggering inflammation by stimulating the immune system through Toll-like receptor 4 [53]. Drugs such as clopidogrel and ticagrelor act indirectly on the gastrointestinal mucosa by inhibiting adenosine diphosphate receptors, reducing the release of vascular endothelial growth factors, inhibiting neovascularization, and hindering gastrointestinal mucosal repair, potentially promoting GIB [54,55]. Furthermore, DAPT has a synergistic effect that can further exacerbate gastrointestinal mucosal damage. OACs increase the risk of GIB through mechanisms such as their local anticoagulation effect, direct corrosive action, and mucosal repair inhibition [56-58]. They prolong clotting time and increase capillary fragility and permeability, thereby elevating the risk of bleeding. Gastrointestinal damage caused by OACs is associated with small intestinal mucosal permeability glycoproteins, which regulate OACs concentration in the gastrointestinal tract. An incompletely absorbed OACs in the gastrointestinal tract may exert a local effect on the mucosa, leading to bleeding [59]. The mechanisms outlined above suggest a potential link, highlighting the need for future experimental studies to further explore the molecular mechanisms underlying the relationship between preoperative medications and GIB risk.

ML technology is often regarded as a “black box,” with its prediction and reasoning processes lacking transparency. Therefore, understanding how ML models make decisions is crucial for clinicians to build trust in the model and identify potential biases. This underscores another key advantage of our study: the use of the SHAP method to provide both global and local explanations of the model [29,60]. In the global explanation, the SHAP summary plot illustrates the overall distribution of each feature’s impact on the model output. Moreover, in GIBCG prediction, the top 5 important admission features include cardiac function–related indicators (such as left ventricular ejection fraction and history of chronic heart failure), age, platelet count, and INR. Notably, left ventricular dysfunction and heart failure are among the most significant independent predictors of mortality and other major adverse events, including bleeding, following CABG [61,62]. The increased risk of postoperative GIB in patients with heart failure may be attributed to reduced cardiac output, which leads to gastrointestinal hypoperfusion, resulting in ischemia and hypoxia of the gastrointestinal mucosa and ultimately weakening its protective barrier function [63]. During ischemia-reperfusion, the release of oxygen-free radicals and inflammatory mediators further exacerbates gastric mucosal injury [64], thereby increasing the risk of ulcers and bleeding. Another important factor is age. Specifically, older adult patients are at a higher risk of GIBCG due to increased vascular fragility [65], impaired gastrointestinal mucosal barrier function [66], multiple comorbidities, and long-term use of gastrointestinal-damaging medications (eg, aspirin). Studies have shown that age over 65 years (OR 2.1) is a risk factor for gastrointestinal complications after CABG [67], while age (OR 1.04) is a predictor of bleeding and transfusion during surgery [68]. These findings align with the interpretation of features in our model, underscoring their clinical significance. Specifically, older adult patients or those with poor preoperative cardiac function are at an increased risk of developing GIB postoperatively and warrant heightened attention. For such populations, greater emphasis should be placed on GIB prevention during preoperative preparation, with enhanced perioperative monitoring, protection of cardiac function, and optimization of blood flow management. Furthermore, coagulation status is closely associated with the occurrence of GIB after cardiac surgery [69-73]. In particular, platelet counts [72] and INR [73] are key indicators of coagulation function that are simple to measure and readily accessible. Abnormal values at admission can help clinicians promptly adjust perioperative antithrombotic treatment strategies, thereby reducing the risk of GIBCG.

Local explanations provide personalized interpretations for each patient’s prediction, while force plots visualize the specific contributions of each feature to the predicted value. For example, in Figure 4, patient 1 is relatively young and has comparatively good cardiac function. However, decreased albumin, elevated white blood cell count, and slightly lower platelet levels (though within the normal range) are identified as the main factors contributing to the increased risk of GIBCG. Previous studies have shown that preoperative hypoalbuminemia is significantly associated with increased mortality [74,75] and postoperative complications, including bleeding [74], in cardiac surgery patients. Notably, an elevated white blood cell count reflects an underlying inflammatory state, which has been associated with increased mortality and complications following CABG [76,77]. Nutritional support, close monitoring of liver function, management of inflammatory responses, and optimization of antithrombotic therapy may help reduce the risk of GIBCG in this patient. Based on readily accessible features, our web platform enables early prediction of GIBCG risk and provides personalized explanations through force plots. This serves as a valuable reference for clinicians in making individualized decisions, thereby supporting the precise prevention of GIBCG.

ML is increasingly applied in personalized medicine and has the potential to improve patient outcomes. However, ethical and practical considerations warrant further discussion [78]. First, patient data privacy is critical. Even deidentified data can be reidentified through available data points [79] or triangulation with other data sets [80]. To mitigate this risk, strategies such as suppression (removing sensitive information), generalization (transforming specific data into ranges), and data minimization (collecting only essential data) are applied [78]. Second, obtaining consent from each patient poses a challenge in acquiring large-scale training data. Alternative methods, such as dynamic consent, broad consent, and implied consent with opt-out options, have been proposed. In our retrospective cohort, broad consent was obtained, allowing for the future use of anonymized data without specific knowledge of the studies [81]. Third, algorithmic bias is a significant concern in ML applications. As ML models are trained on historical data, any biases present in the training data (eg, related to race, gender, or socioeconomic status) may be perpetuated or even amplified in predictions [82]. In addition, experts involved in labeling the data may inadvertently introduce their own biases into the algorithm [83]. Addressing algorithmic bias requires ensuring the diversity and representativeness of the training data set. For instance, our study utilized multicenter data for model training. Moreover, enhancing model interpretability allows health care professionals to understand its decision-making process, enabling them to identify and mitigate potential biases. Continuous monitoring and evaluation should also be conducted during the model’s application. In future research, we plan to validate our model across different populations and refine it based on feedback [84]. Notably, surveys indicate that many American adults are uncomfortable with doctors relying on ML for decision-making [85]. Most ML systems are designed to assist clinicians in decision-making with oversight rather than functioning autonomously [86]. This approach helps alleviate public concerns about ML-driven decisions while ensuring health care quality and patient safety. Future efforts to integrate ML into health care should prioritize the development and implementation of standardized ethical frameworks and guidelines.

Recently, ML has made significant progress in the field of cardiovascular diseases and related surgeries [17,18]. Research has primarily focused on surgical risk assessment [87], prediction of postoperative complications [88-93], evaluation of patient prognosis [94,95], and guidance for personalized medicine [96]. Several ML models have been developed to predict postoperative complications in cardiac surgery, including bleeding [88,89], acute kidney injury [90,91], delirium [92], and major adverse cardiovascular and cerebrovascular events [93]. Furthermore, studies have shown that ML algorithms perform exceptionally well in predicting severe bleeding after CABG [89]. Additionally, ML has been demonstrated to outperform traditional risk-scoring methods in predicting the risk of GIB following antithrombotic therapy [20]. However, in the surgical field, ML models specifically targeting GIBCG remain unavailable. To date, only a single-center study has proposed a traditional risk prediction model [10], which does not enable preoperative prediction of GIBCG risk, thereby limiting early prevention efforts. To address this critical gap, we developed an ML model using multicenter data to predict the risk of GIBCG. The model was validated on 2 independent external cohorts, demonstrating its ability to accurately predict a patient’s risk of GIBCG upon admission and guide personalized preventive measures. This approach may help reduce the incidence of this complication and improve patient outcomes. Furthermore, it highlights the significant value of ML in cardiovascular surgery for preventing complications and enhancing the quality of patient care.

### Limitations

Our study has several limitations. First, the inclusion of retrospective cohorts may introduce selection bias. However, incorporating a prospective cohort in the model development, along with the use of rigorous selection criteria and a substantial sample size, may help mitigate this limitation. Second, the model was developed based on a Chinese population, and its generalizability to global populations remains uncertain. Nevertheless, its acceptable performance in critical care cohorts in the United States suggests its potential broader applicability. Future prospective validation is necessary to further refine and evaluate the model’s performance. Third, the causal relationships between predictive features (risk factors) and GIBCG, as well as between preoperative medication and GIBCG, require further investigation. Randomized controlled trials are essential to determine whether interventions targeting these risk factors and modifications to preoperative medication can effectively prevent GIBCG. Lastly, this study was retrospectively registered, which may introduce biases in design and reporting. To address this, we ensured transparency by adhering to strict inclusion and exclusion criteria and providing a detailed methodology and results. The substantial sample size and multicenter design help mitigate potential biases from retrospective registration and enhance the study’s reliability.

### Conclusions

In this study, we successfully developed an XGBoost-based ML model for GIBCG risk prediction using 15 readily accessible admission features, facilitating precision prevention and personalized management of GIBCG. For high-risk patients identified by the model—who have an increased risk of in-hospital mortality and require heightened clinical attention—prophylactic PPIs administration and adjustments to antithrombotic therapy may help reduce the incidence of GIBCG. Moreover, the model demonstrated consistent and excellent performance across both the derivation and validation cohorts, confirming its robustness and scalability. To enhance clinical applicability, it has also been integrated into a web-based risk prediction calculator.

## Appendix

Multimedia Appendix 1 Additional analysis.

## Abbreviations

AUROC: area under the receiver operating characteristic curve
CABG: coronary artery bypass grafting
COX: cyclooxygenase
DAPT: dual antiplatelet therapy
DCA: decision curve analysis
GIB: gastrointestinal bleeding
GIBCG: gastrointestinal bleeding after coronary artery bypass grafting
ICU: intensive care unit
INR: international normalized ratio
K-Best: K-best feature selection
LASSO: least absolute shrinkage and selection operator
MIMIC-IV: Medical Information Mart for Intensive Care IV
ML: machine learning
OACs: oral anticoagulants
OR: odds ratio
PPIs: proton pump inhibitors
PRECISE-DAPT: predicting bleeding complications in patients undergoing stent implantation and subsequent dual antiplatelet therapy
RFE: random forest recursive feature elimination
SAPT: single antiplatelet therapy
SHAP: Shapley Additive Explanations
SOFA: Sequential Organ Failure Assessment
XGBoost: Extreme Gradient Boosting

## Data Availability

Researchers interested in data access should contact the corresponding author (ZJ). Data requests will need to undergo ethical and legal approval by the relevant institutions. The MIMIC-IV data we used are available at [97]. All the codes used for extraction, cleaning, filtering, and modeling are available on GitHub [98].

## References

[1] Vervoort D, Lee G, Ghandour H, Guetter CR, Adreak N, Till BM, Lin Y (2024). Global cardiac surgical volume and gaps: trends, targets, and way forward. Ann Thorac Surg Short Rep.

[2] Alkhouli M, Alqahtani F, Kalra A, Gafoor S, Alhajji M, Alreshidan M, Holmes DR, Lerman A (2020). Trends in characteristics and outcomes of patients undergoing coronary revascularization in the United States, 2003-2016. JAMA Netw Open.

[3] Marsoner K, Voetsch A, Lierzer C, Sodeck GH, Fruhwald S, Dapunt O, Mischinger HJ, Kornprat P (2019). Gastrointestinal complications following on-pump cardiac surgery-a propensity matched analysis. PLoS One.

[4] Elgharably Haytham, Gamaleldin Maysoon, Ayyat Kamal S, Zaki Anthony, Hodges Kevin, Kindzelski Bogdan, Sharma Shashank, Hassab Tarek, Yongue Camille, Serna Solanus de la, Perez Juan, Spencer Capri, Bakaeen Faisal G, Steele Scott R, Gillinov A Marc, Svensson Lars G, Pettersson Gosta B (2021). Serious gastrointestinal complications after cardiac surgery and associated mortality. Ann Thorac Surg.

[5] Chor CYT, Mahmood S, Khan IH, Shirke M, Harky A (2020). Gastrointestinal complications following cardiac surgery. Asian Cardiovasc Thorac Ann.

[6] Rodriguez R, Robich M, Plate J, Trooskin Stanley Z, Sellke Frank W (2010). Gastrointestinal complications following cardiac surgery: a comprehensive review. J Card Surg.

[7] Chaudhry R, Zaki J, Wegner R, Pednekar G, Tse A, Sheinbaum R, Williams GW (2017). Gastrointestinal complications after cardiac surgery: a nationwide population-based analysis of morbidity and mortality predictors. J Cardiothorac Vasc Anesth.

[8] Hess NR, Seese LM, Hong Y, Afflu D, Wang Y, Thoma FW, Kilic A (2021). Gastrointestinal complications after cardiac surgery: incidence, predictors, and impact on outcomes. J Card Surg.

[9] Wolska N, Rozalski M (2019). Blood platelet adenosine receptors as potential targets for anti-platelet therapy. Int J Mol Sci.

[10] Yang M, Zhan S, Gao H, Liao C, Li S (2023). Construction and validation of risk prediction model for gastrointestinal bleeding in patients after coronary artery bypass grafting. Sci Rep.

[11] Wang X, Dong B, Hong B, Gong Y, Wang W, Wang J, Zhou Z, Jiang W (2017). Long-term prognosis in patients continuing taking antithrombotics after peptic ulcer bleeding. World J Gastroenterol.

[12] Costa F, van Klaveren D, James S, Heg D, Räber Lorenz, Feres F, Pilgrim T, Hong M, Kim H, Colombo A, Steg PG, Zanchin T, Palmerini T, Wallentin L, Bhatt DL, Stone GW, Windecker S, Steyerberg EW, Valgimigli M, PRECISE-DAPT Study Investigators (2017). Derivation and validation of the predicting bleeding complications in patients undergoing stent implantation and subsequent dual antiplatelet therapy (PRECISE-DAPT) score: a pooled analysis of individual-patient datasets from clinical trials. Lancet.

[13] Krawiec F, Maitland A, Duan Q, Faris P, Belletrutti PJ, Kent WD (2017). Duodenal ulcers are a major cause of gastrointestinal bleeding after cardiac surgery. J Thorac Cardiovasc Surg.

[14] Greener JG, Kandathil SM, Moffat L, Jones DT (2022). A guide to machine learning for biologists. Nat Rev Mol Cell Biol.

[15] Haug CJ, Drazen JM (2023). Artificial intelligence and machine learning in clinical medicine, 2023. N Engl J Med.

[16] Rajkomar A, Dean J, Kohane I (2019). Machine learning in medicine. N Engl J Med.

[17] Johnson KW, Torres Soto J, Glicksberg BS, Shameer K, Miotto R, Ali M, Ashley E, Dudley JT (2018). Artificial intelligence in cardiology. J Am Coll Cardiol.

[18] El Sherbini A, Rosenson RS, Al Rifai M, Virk HUH, Wang Z, Virani S, Glicksberg BS, Lavie CJ, Krittanawong C (2024). Artificial intelligence in preventive cardiology. Prog Cardiovasc Dis.

[19] Komuro J, Kusumoto D, Hashimoto H, Yuasa S (2023). Machine learning in cardiology: clinical application and basic research. J Cardiol.

[20] Herrin J, Abraham NS, Yao X, Noseworthy PA, Inselman J, Shah ND, Ngufor C (2021). Comparative effectiveness of machine learning approaches for predicting gastrointestinal bleeds in patients receiving antithrombotic treatment. JAMA Netw Open.

[21] Johnson AEW, Bulgarelli L, Shen L, Gayles A, Shammout A, Horng S, Pollard TJ, Hao S, Moody B, Gow B, Lehman LH, Celi LA, Mark RG (2023). MIMIC-IV, a freely accessible electronic health record dataset. Sci Data.

[22] Riley RD, Ensor J, Snell KIE, Harrell FE, Martin GP, Reitsma JB, Moons KGM, Collins G, van Smeden M (2020). Calculating the sample size required for developing a clinical prediction model. BMJ.

[23] Tokar JL, Higa JT (2022). Acute gastrointestinal bleeding. Ann Intern Med.

[24] Raju GS, Gerson L, Das A, Lewis B, American Gastroenterological Association (2007). American Gastroenterological Association (AGA) Institute medical position statement on obscure gastrointestinal bleeding. Gastroenterology.

[25] Rockey DC, Altayar O, Falck-Ytter Y, Kalmaz D (2020). AGA technical review on gastrointestinal evaluation of iron deficiency anemia. Gastroenterology.

[26] Pudjihartono N, Fadason T, Kempa-Liehr AW, O'Sullivan JM (2022). A review of feature selection methods for machine learning-based disease risk prediction. Front Bioinform.

[27] Rufibach K (2010). Use of Brier score to assess binary predictions. J Clin Epidemiol.

[28] Zhao L, Leng Y, Hu Y, Xiao J, Li Q, Liu C, Mao Y (2024). Understanding decision curve analysis in clinical prediction model research. Postgrad Med J.

[29] Lundberg SM, Erion G, Chen H, DeGrave A, Prutkin JM, Nair B, Katz R, Himmelfarb J, Bansal N, Lee S (2020). From local explanations to global understanding with explainable AI for trees. Nat Mach Intell.

[30] Parsa AB, Movahedi A, Taghipour H, Derrible S, Mohammadian A( (2020). Toward safer highways, application of XGBoost and SHAP for real-time accident detection and feature analysis. Accid Anal Prev.

[31] Calculation tool for predicting gastrointestinal bleeding after coronary artery bypass grafting. XSmart Analysis.

[32] Halling CMB, Møller Morten Hylander, Marker S, Krag M, Kjellberg J, Perner A, Gyrd-Hansen D (2022). The effects of pantoprazole vs. placebo on 1-year outcomes, resource use and employment status in ICU patients at risk for gastrointestinal bleeding: a secondary analysis of the SUP-ICU trial. Intensive Care Med.

[33] MacLaren R, Dionne J, Granholm A, Alhazzani Waleed, Szumita Paul M, Olsen Keith, Barletta Jeffrey F, Møller Morten Hylander, Karvellas Constantine J, Wischmeyer Paul, DePriest Ashley, Carlos Victor, Argetsinger Debora, Carothers John J, Lee Rosemary, Napolitano Lena, Perri Dan, Naylor Douglas F (2024). Society of Critical Care Medicine and American Society of Health-System Pharmacists guideline for the prevention of stress-related gastrointestinal bleeding in critically ill adults. Crit Care Med.

[34] Elshaer A, Abraham NS (2024). Management of anticoagulant and antiplatelet agents in acute gastrointestinal bleeding and prevention of gastrointestinal bleeding. Gastrointest Endosc Clin N Am.

[35] Sousa-Uva M, Milojevic Milan, Head Stuart J, Jeppsson Anders (2018). The 2017 EACTS guidelines on perioperative medication in adult cardiac surgery and patient blood management. Eur J Cardiothorac Surg.

[36] Gralnek IM, Stanley AJ, Morris AJ, Camus M, Lau J, Lanas A, Laursen SB, Radaelli F, Papanikolaou IS, Cúrdia Gonçalves Tiago, Dinis-Ribeiro M, Awadie H, Braun G, de Groot N, Udd M, Sanchez-Yague A, Neeman Z, van Hooft JE (2021). Endoscopic diagnosis and management of nonvariceal upper gastrointestinal hemorrhage (NVUGIH): European Society of Gastrointestinal Endoscopy (ESGE) Guideline - Update 2021. Endoscopy.

[37] Oakland K, Chadwick G, East JE, Guy R, Humphries A, Jairath V, McPherson S, Metzner M, Morris AJ, Murphy MF, Tham T, Uberoi R, Veitch AM, Wheeler J, Regan C, Hoare J (2019). Diagnosis and management of acute lower gastrointestinal bleeding: guidelines from the British Society of Gastroenterology. Gut.

[38] Kamada T, Satoh K, Itoh T, Ito M, Iwamoto J, Okimoto T, Kanno T, Sugimoto M, Chiba T, Nomura S, Mieda M, Hiraishi H, Yoshino J, Takagi A, Watanabe S, Koike K (2021). Evidence-based clinical practice guidelines for peptic ulcer disease 2020. J Gastroenterol.

[39] Kulik A, Ruel M, Jneid H, Ferguson TB, Hiratzka LF, Ikonomidis JS, Lopez-Jimenez F, McNallan SM, Patel M, Roger VL, Sellke FW, Sica DA, Zimmerman L (2015). Secondary prevention after coronary artery bypass graft surgery. Circulation.

[40] Jacob M, Smedira N, Blackstone E, Williams S, Cho L (2011). Effect of timing of chronic preoperative aspirin discontinuation on morbidity and mortality in coronary artery bypass surgery. Circulation.

[41] Fox KA, Mehta SR, Peters R, Zhao F, Lakkis N, Gersh BJ, Yusuf S, Clopidogrel in Unstable angina to prevent Recurrent ischemic Events Trial (2004). Benefits and risks of the combination of clopidogrel and aspirin in patients undergoing surgical revascularization for non-ST-elevation acute coronary syndrome: the Clopidogrel in Unstable angina to prevent Recurrent ischemic Events (CURE) Trial. Circulation.

[42] McLean DS, Sabatine MS, Guo W, McCabe CH, Cannon CP (2007). Benefits and risks of clopidogrel pretreatment before coronary artery bypass grafting in patients with ST-elevation myocardial infarction treated with fibrinolytics in CLARITY-TIMI 28. J Thromb Thrombolysis.

[43] Veitch AM, Radaelli F, Alikhan R, Dumonceau JM, Eaton D, Jerrome J, Lester W, Nylander D, Thoufeeq M, Vanbiervliet G, Wilkinson JR, Van Hooft JE (2021). Endoscopy in patients on antiplatelet or anticoagulant therapy: British Society of Gastroenterology (BSG) and European Society of Gastrointestinal Endoscopy (ESGE) guideline update. Gut.

[44] Abraham NS, Noseworthy PA, Inselman J, Herrin J, Yao X, Sangaralingham LR, Cornish G, Ngufor C, Shah ND (2020). Risk of gastrointestinal bleeding increases with combinations of antithrombotic agents and patient age. Clin Gastroenterol Hepatol.

[45] Yang Y, Metz DC (2010). Safety of proton pump inhibitor exposure. Gastroenterology.

[46] Patel P, Sengupta N (2020). PPIs and beyond: a framework for managing anticoagulation-related gastrointestinal bleeding in the era of COVID-19. Dig Dis Sci.

[47] Guo H, Ye Z, Huang R (2021). Clinical outcomes of concomitant use of proton pump inhibitors and dual antiplatelet therapy: a systematic review and meta-analysis. Front Pharmacol.

[48] Kherad O, Restellini S, Martel M, Barkun A (2019). Proton pump inhibitors for upper gastrointestinal bleeding. Best Pract Res Clin Gastroenterol.

[49] Yu Z, He J, Cao R, Yang Z, Li B, Hong J, Chen Y, Zhu L (2023). Proton pump inhibitor has no effect in the prevention of post-endoscopic sphincterotomy delayed bleeding: a prospective randomized controlled trial. Front Med (Lausanne).

[50] Goddard PJ, Hills BA, Lichtenberger LM (1987). Does aspirin damage canine gastric mucosa by reducing its surface hydrophobicity?. Am J Physiol.

[51] Hernández Carlos, Barrachina MD, Vallecillo-Hernández Jorge, Álvarez Ángeles, Ortiz-Masiá Dolores, Cosín-Roger Jesús, Esplugues JV, Calatayud S (2016). Aspirin-induced gastrointestinal damage is associated with an inhibition of epithelial cell autophagy. J Gastroenterol.

[52] Zhang W, Wang M, Hua G, Li Q, Wang X, Lang R, Weng W, Xue C, Zhu B (2021). Inhibition of aspirin-induced gastrointestinal injury: systematic review and network meta-analysis. Front Pharmacol.

[53] Otani K, Tanigawa T, Watanabe T, Shimada S, Nadatani Y, Nagami Y, Tanaka F, Kamata N, Yamagami H, Shiba M, Tominaga K, Fujiwara Y, Arakawa T (2017). Microbiota plays a key role in non-steroidal anti-inflammatory drug-induced small intestinal damage. Digestion.

[54] Bhatt D, Scheiman J, Abraham N, Antman Em, Chan Fk, Furberg Cd, Johnson Da, Mahaffey Kw, Quigley Em, Harrington Robert A, Bates Eric R, Bridges Charles R, Eisenberg Mark J, Ferrari Victor A, Hlatky Mark A, Kaul Sanjay, Lindner Jonathan R, Moliterno David J, Mukherjee Debabrata, Schofield Richard S, Rosenson Robert S, Stein James H, Weitz Howard H, Wesley Deborah J, American College of Cardiology Foundation Task Force on Clinical Expert Consensus Documents (2008). ACCF/ACG/AHA 2008 expert consensus document on reducing the gastrointestinal risks of antiplatelet therapy and NSAID use: a report of the American College of Cardiology Foundation Task Force on Clinical Expert Consensus Documents. J Am Coll Cardiol.

[55] Kou N, Xue M, Yang L, Zang Ming-Xuan, Qu Hua, Wang Ming-Ming, Miao Yu, Yang Bin, Shi Da-Zhuo (2018). Panax quinquefolius saponins combined with dual antiplatelet drug therapy alleviate gastric mucosal injury and thrombogenesis through the COX/PG pathway in a rat model of acute myocardial infarction. PLoS One.

[56] Cheung K, Leung WK (2017). Gastrointestinal bleeding in patients on novel oral anticoagulants: risk, prevention and management. World J Gastroenterol.

[57] Desai J, Kolb J, Weitz J, Aisenberg J (2013). Gastrointestinal bleeding with the new oral anticoagulants--defining the issues and the management strategies. Thromb Haemost.

[58] Thapa N, Shatzel J, Deloughery TG, Olson SR (2019). Direct oral anticoagulants in gastrointestinal malignancies: is the convenience worth the risk?. J Gastrointest Oncol.

[59] Blech S, Ebner T, Ludwig-Schwellinger E, Stangier J, Roth W (2008). The metabolism and disposition of the oral direct thrombin inhibitor, dabigatran, in humans. Drug Metab Dispos.

[60] Ge S, Chen J, Wang W, Zhang L, Teng Y, Yang C, Wang H, Tao Y, Chen Z, Li R, Niu Y, Zuo C, Tan L (2024). Predicting who has delayed cerebral ischemia after aneurysmal subarachnoid hemorrhage using machine learning approach: a multicenter, retrospective cohort study. BMC Neurol.

[61] Fortescue EB, Kahn K, Bates DW (2001). Development and validation of a clinical prediction rule for major adverse outcomes in coronary bypass grafting. Am J Cardiol.

[62] Yau TM, Fedak PW, Weisel RD, Teng C, Ivanov J (1999). Predictors of operative risk for coronary bypass operations in patients with left ventricular dysfunction. J Thorac Cardiovasc Surg.

[63] Polsinelli VB, Sinha A, Shah SJ (2017). Visceral congestion in heart failure: right ventricular dysfunction, splanchnic hemodynamics, and the intestinal microenvironment. Curr Heart Fail Rep.

[64] Bhattacharyya A, Chattopadhyay R, Mitra S, Crowe SE (2014). Oxidative stress: an essential factor in the pathogenesis of gastrointestinal mucosal diseases. Physiol Rev.

[65] Lakatta EG, Levy D (2003). Arterial and cardiac aging: major shareholders in cardiovascular disease enterprises: Part I: aging arteries: a "set up" for vascular disease. Circulation.

[66] Tatge L, Solano Fonseca R, Douglas PM (2023). A framework for intestinal barrier dysfunction in aging. Nat Aging.

[67] Rodriguez F, Nguyen T, Galanko J, Morton John (2007). Gastrointestinal complications after coronary artery bypass grafting: a national study of morbidity and mortality predictors. J Am Coll Surg.

[68] Karkouti K, Cohen MM, McCluskey SA, Sher GD (2001). A multivariable model for predicting the need for blood transfusion in patients undergoing first-time elective coronary bypass graft surgery. Transfusion.

[69] Karkouti K, McCluskey S, Syed S, Pazaratz Chris, Poonawala Humara, Crowther Mark A (2010). The influence of perioperative coagulation status on postoperative blood loss in complex cardiac surgery: a prospective observational study. Anesth Analg.

[70] Li X, Wang R, Sun D, Yao Y, Wang T, Luo G, Liu M, Xu J, Cheng Z, Gao Q, Wang Y, Wu C, Xu G, Lv T, Zou J, Yan M (2023). Risk factors for hypocoagulability after cardiac surgery: a retrospective study. Clin Appl Thromb Hemost.

[71] Bartoszko J, Wijeysundera D, Karkouti K, Callum Jeannie, Rao Vivek, Crowther Mark, Grocott Hilary P, Pinto Ruxandra, Scales Damon C, Achen Blaine, Brar Sukhpal, Morrison Doug, Wong David, Bussières Jean S, de Waal Tonya, Harle Christopher, de Médicis Étienne, McAdams Charles, Syed Summer, Tran Diem, Waters Terry, Transfusion Avoidance in Cardiac Surgery Study Investigators (2018). Comparison of two major perioperative bleeding scores for cardiac surgery trials: universal definition of perioperative bleeding in cardiac surgery and European coronary artery bypass grafting bleeding severity grade. Anesthesiology.

[72] Ozyilmaz I, Öztürk Erkut, Ozalp S, Recep BZT, Hatemi AC, Tanıdır (2024). Assessment of the frequency and risk factors of gastrointestinal bleeding after cardiopulmonary bypass in paediatric cases. Cardiol Young.

[73] Wu S, Lv M, Ma F, Feilong Z, Fang G, Zhang J (2023). A new model (Alfalfa-Warfarin-GIB) for predicting the risk of major gastrointestinal bleeding in warfarin patients. Eur J Clin Pharmacol.

[74] Xu R, Hao M, Zhou W, Liu M, Wei Y, Xu J, Zhang W (2023). Preoperative hypoalbuminemia in patients undergoing cardiac surgery: a meta-analysis. Surg Today.

[75] de la Cruz KI, Bakaeen FG, Wang XL, Huh J, LeMaire SA, Coselli JS, Chu D (2011). Hypoalbuminemia and long-term survival after coronary artery bypass: a propensity score analysis. Ann Thorac Surg.

[76] Aizenshtein A, Kachel E, Liza G, Hijazi Basem, Blum Arnon (2020). Effects of preoperative WBC count on post-CABG surgery clinical outcome. South Med J.

[77] Newall N, Grayson AD, Oo AY, Palmer ND, Dihmis WC, Rashid A, Stables RH (2006). Preoperative white blood cell count is independently associated with higher perioperative cardiac enzyme release and increased 1-year mortality after coronary artery bypass grafting. Ann Thorac Surg.

[78] Aggarwal N, Singh A, Garcia P, Guha S (2024). Ethical implications of artificial intelligence in gastroenterology. Clin Gastroenterol Hepatol.

[79] Na L, Yang C, Lo C, Zhao F, Fukuoka Y, Aswani A (2018). Feasibility of reidentifying individuals in large national physical activity data sets from which protected health information has been removed with use of machine learning. JAMA Netw Open.

[80] Xiang D, Cai W (2021). Privacy protection and secondary use of health data: strategies and methods. Biomed Res Int.

[81] van Delden JJM, van der Graaf R (2017). Revised CIOMS international ethical guidelines for health-related research involving humans. JAMA.

[82] Rashid D, Hirani R, Khessib S, Ali N, Etienne M (2024). Unveiling biases of artificial intelligence in healthcare: navigating the promise and pitfalls. Injury.

[83] Acerbi A, Stubbersfield JM (2023). Large language models show human-like content biases in transmission chain experiments. Proc Natl Acad Sci U S A.

[84] Lin S, Pandit S, Tritsch T, Levy A, Shoja M (2024). What goes in, must come out: generative artificial intelligence does not present algorithmic bias across race and gender in medical residency specialties. Cureus.

[85] Tyson A, Pasquini G, Spencer A (2023). 60% of Americans would be uncomfortable with provider relying on AI in their own health care. Pew Research Center.

[86] Topol EJ (2019). High-performance medicine: the convergence of human and artificial intelligence. Nat Med.

[87] Shahian DM, Lippmann RP (2022). Commentary: Machine learning and cardiac surgery risk prediction. J Thorac Cardiovasc Surg.

[88] Hui V, Litton E, Edibam C, Geldenhuys Agneta, Hahn Rebecca, Larbalestier Robert, Wright Brian, Pavey Warren (2023). Using machine learning to predict bleeding after cardiac surgery. Eur J Cardiothorac Surg.

[89] Gao Y, Liu X, Wang L, Wang S, Yu Y, Ding Y, Wang J, Ao H (2022). Machine learning algorithms to predict major bleeding after isolated coronary artery bypass grafting. Front Cardiovasc Med.

[90] Luo X, Kang Y, Duan S, Yan P, Song G, Zhang N, Yang S, Li J, Zhang H (2023). Machine learning-based prediction of acute kidney injury following pediatric cardiac surgery: model development and validation study. J Med Internet Res.

[91] Song Y, Zhai W, Ma S, Wu Y, Ren M, Van den Eynde J, Nardi P, Pang PYK, Ali JM, Han J, Guo Z (2024). Machine learning-based prediction of off-pump coronary artery bypass grafting-associated acute kidney injury. J Thorac Dis.

[92] Yang T, Yang H, Liu Y, Liu X, Ding Y, Li R, Mao A, Huang Y, Li X, Zhang Y, Yu F (2024). Postoperative delirium prediction after cardiac surgery using machine learning models. Comput Biol Med.

[93] Mahajan A, Esper S, Oo TH, McKibben J, Garver M, Artman J, Klahre C, Ryan J, Sadhasivam S, Holder-Murray J, Marroquin OC (2023). Development and validation of a machine learning model to identify patients before surgery at high risk for postoperative adverse events. JAMA Netw Open.

[94] Tong C, Du X, Chen Y, Zhang Kan, Shan Mengqin, Shen Ziyun, Zhang Haibo, Zheng Jijian (2024). Machine learning prediction model of major adverse outcomes after pediatric congenital heart surgery: a retrospective cohort study. Int J Surg.

[95] Castela Forte J, Yeshmagambetova G, van der Grinten ML, Scheeren TWL, Nijsten MWN, Mariani MA, Henning RH, Epema AH (2022). Comparison of machine learning models including preoperative, intraoperative, and postoperative data and mortality after cardiac surgery. JAMA Netw Open.

[96] Xue L, He S, Singla R, Qin Qiong, Ding Yinglong, Liu Linsheng, Ding Xiaoliang, Bediaga-Bañeres Harbil, Arrasate Sonia, Durado-Sanchez Aliuska, Zhang Yuzhen, Shen Zhenya, Shen Bairong, Miao Liyan, González-Díaz Humberto (2024). Machine learning guided prediction of warfarin blood levels for personalized medicine based on clinical longitudinal data from cardiac surgery patients: a prospective observational study. Int J Surg.

[97] PhysioNet.

[98] GitHub.

