# Supplementary material for: Machine Learning Models With Prognostic Implications for Predicting Gastrointestinal Bleeding After Coronary Artery Bypass Grafting and Guiding Personalized Medicine: Multicenter Cohort Study
Source: J Med Internet Res. 2025 Mar 6;27:e68509. doi: 10.2196/68509 (PMC11926454; doi:10.2196/68509)
Supplement: Multimedia Appendix 1 [file jmir_v27i1e68509_app1.doc]

**Supplementary materials**

[Figure S1. Flow Diagram of Participant Selection 3](#__RefHeading___Toc4298)

[Figure S2. Proportion of Patients with Different Degrees of Gastrointestinal Bleeding Among Those with GIBCG Across Study Cohorts 4](#__RefHeading___Toc7322)

[Figure S3. Receiver Operating Characteristic Curves of the Model for Predicting Severe Gastrointestinal Bleeding or Gastrointestinal Bleeding 5](#__RefHeading___Toc20689)

[Figure S4. Changes in Model AUROC Across Subsets of Patients with Varying Disease Severity in the MIMIC Cohort 6](#__RefHeading___Toc17763)

[Figure S5. Comparison of In-Hospital Mortality Between High-Risk and Low-Risk Populations Identified by the Model 7](#__RefHeading___Toc1925)

[Figure S6. Receiver Operating Characteristic Curves of the Model and the PRECISE-DAPT Score for Predicting In-hospital Mortality. 8](#__RefHeading___Toc31588)

[Table S1. The Calculation Process, Formula, and Results of Sample Size Determination. 8](#__RefHeading___Toc12023)

[Table S2. Admission Features for Machine Learning Models 10](#__RefHeading___Toc12574)

[Table S3. ICD-9/ICD-10 Codes are Used to Identify Chronic Comorbidities and Procedures 12](#__RefHeading___Toc13495)

[Table S4. Description of the Data Types and Missing Values for Each Feature 15](#__RefHeading___Toc10673)

[Table S5. Model Hyperparameter Settings, Ranges, and Rationale 16](#__RefHeading___Toc12712)

[Table S6. Baseline Characteristics of Patients with and without Gastrointestinal Bleeding after Coronary Artery Bypass Grafting in the Derivation Cohort 17](#__RefHeading___Toc29317)

[Table S7. Baseline Characteristics of Patients with and without Gastrointestinal Bleeding after Coronary Artery Bypass Grafting in the Drum Tower Validation Cohort 19](#__RefHeading___Toc15703)

[Table S8. Baseline Characteristics of Patients with and without Gastrointestinal Bleeding after Coronary Artery Bypass Grafting in the MIMIC Validation Cohort 21](#__RefHeading___Toc11667)

[Table S9. Results of Five Feature Selection Methods 23](#__RefHeading___Toc16373)

[Table S10. Optimal Hyperparameters for Each Model Configuration 24](#__RefHeading___Toc8190)

[Table S11. Average AUROC and Its 95% Confidence Interval for Each Model in the Training Set (Comprising Four Parts of the Data from the Five-fold Cross-validation Conducted on the Derivation Cohort) 25](#__RefHeading___Toc14596)

[Table S12. Average AUROC and Its 95% Confidence Interval for Each Model in the Internal Validation Set (Comprising the Remaining One Part of Data from the Five-fold Cross-validation) 26](#__RefHeading___Toc1667)

[Table S13. Mean Value of Brier Scores and Its 95% Confidence Interval for Each Model in the Training Set 27](#__RefHeading___Toc18567)

[Table S14. Mean Value of Brier Scores and Its 95% Confidence Interval for Each Model in the Internal Validation Set 28](#__RefHeading___Toc26211)

[Table S15. Univariate and Multivariate Analysis of Risk Factors for Mortality in Patients Across All Cohorts 29](#__RefHeading___Toc30403)

[Table S16. Univariate analysis of preoperative medications in model-differentiated high- and low-risk populations 30](#__RefHeading___Toc1721)

[Table S17. Univariate and Multivariate Analysis of Risk Factors for Gastrointestinal Bleeding after Coronary Artery Bypass Grafting in the High-Risk Subgroup of the Derivation Cohort 31](#__RefHeading___Toc22210)

[Table S18. Univariate and Multivariate Analysis of Risk Factors for Gastrointestinal Bleeding after Coronary Artery Bypass Grafting in the High-Risk Subgroup of the Drum Tower Validation Cohort 33](#__RefHeading___Toc23894)

[Table S19. Univariate and Multivariate Analysis of Risk Factors for Gastrointestinal Bleeding after Coronary Artery Bypass Grafting in the High-Risk Subgroup of the MIMIC Validation Cohort 35](#__RefHeading___Toc16956)

**Figure S1. Flow Diagram of Participant Selection**





* From an initial cohort of 18,938 patients, 2,498 were excluded due to preoperative diagnoses of gastrointestinal bleeding (GIB) or unidentified bleeding causes attributed to comorbidities such as severe liver disease or gastrointestinal malignancy. Specifically, from Anzhen, Luhe, and Chaoyang Hospitals, 2,226 of 13,399 participants (including 14 with severe liver disease, 42 with gastrointestinal malignant tumors, and 2,170 with confirmed preoperative GIB) were excluded. From Drum Tower Hospital, 225 of 2,970 participants (3 with severe liver disease, 12 with gastrointestinal malignant tumors, and 210 with confirmed preoperative GIB) were excluded. From the MIMIC IV database, 47 out of 343 participants ( 1 with severe liver disease, 4 with gastrointestinal malignant tumors, and 42 with confirmed preoperative GIB) were excluded. The remaining 13,399 patients from the three hospitals formed the derivation cohort for the machine learning model construction with five-fold cross-validation. Meanwhile, 2,745 patients from Drum Tower Hospital and 296 from the MIMIC IV database were used for external validation and performance evaluation.

Abbreviations: GI, gastrointestinal; GIB, gastrointestinal bleeding; ML, machine learning

**Figure S2. Proportion of Patients with Different Degrees of Gastrointestinal Bleeding Among Those with GIBCG Across Study Cohorts**


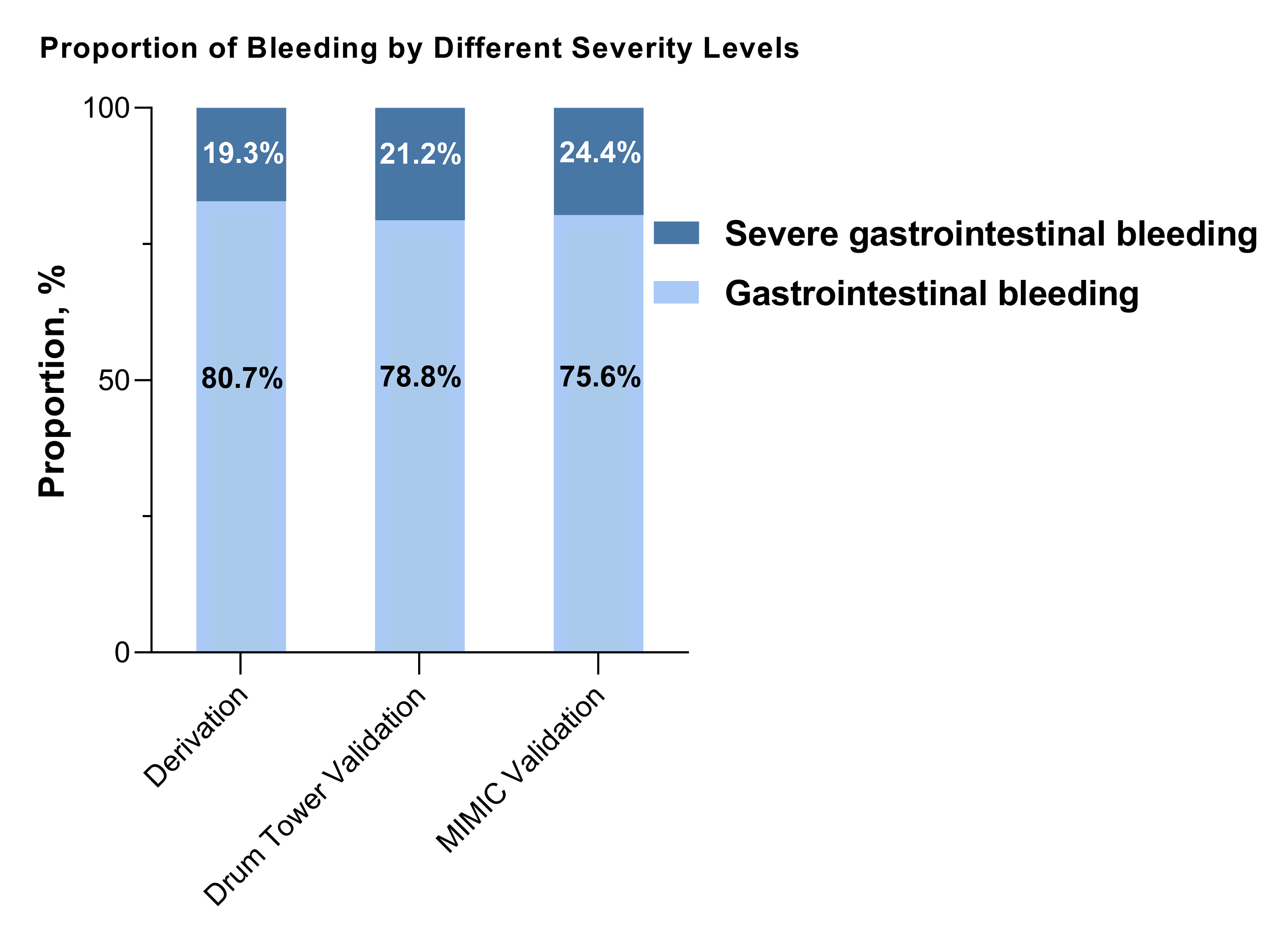


# Figure S3. Receiver Operating Characteristic Curves of the Model for Predicting Severe Gastrointestinal Bleeding or Gastrointestinal Bleeding


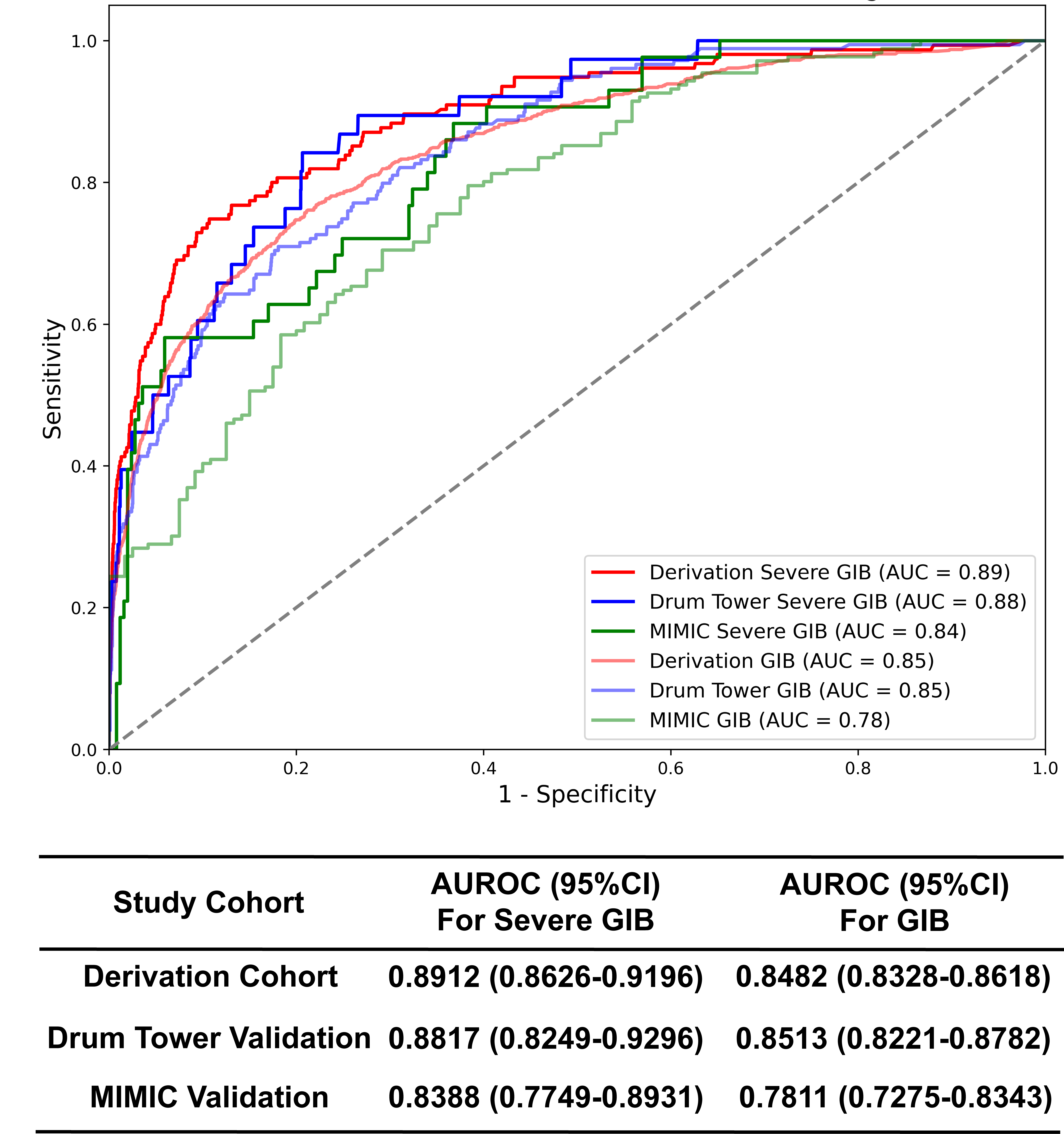


* The solid line represents the prediction of Severe Gastrointestinal Bleeding, while the transparent line represents the prediction of all Gastrointestinal Bleeding.

**Figure S4. Changes in Model AUROC Across Subsets of Patients with Varying Disease Severity in the MIMIC Cohort**

**
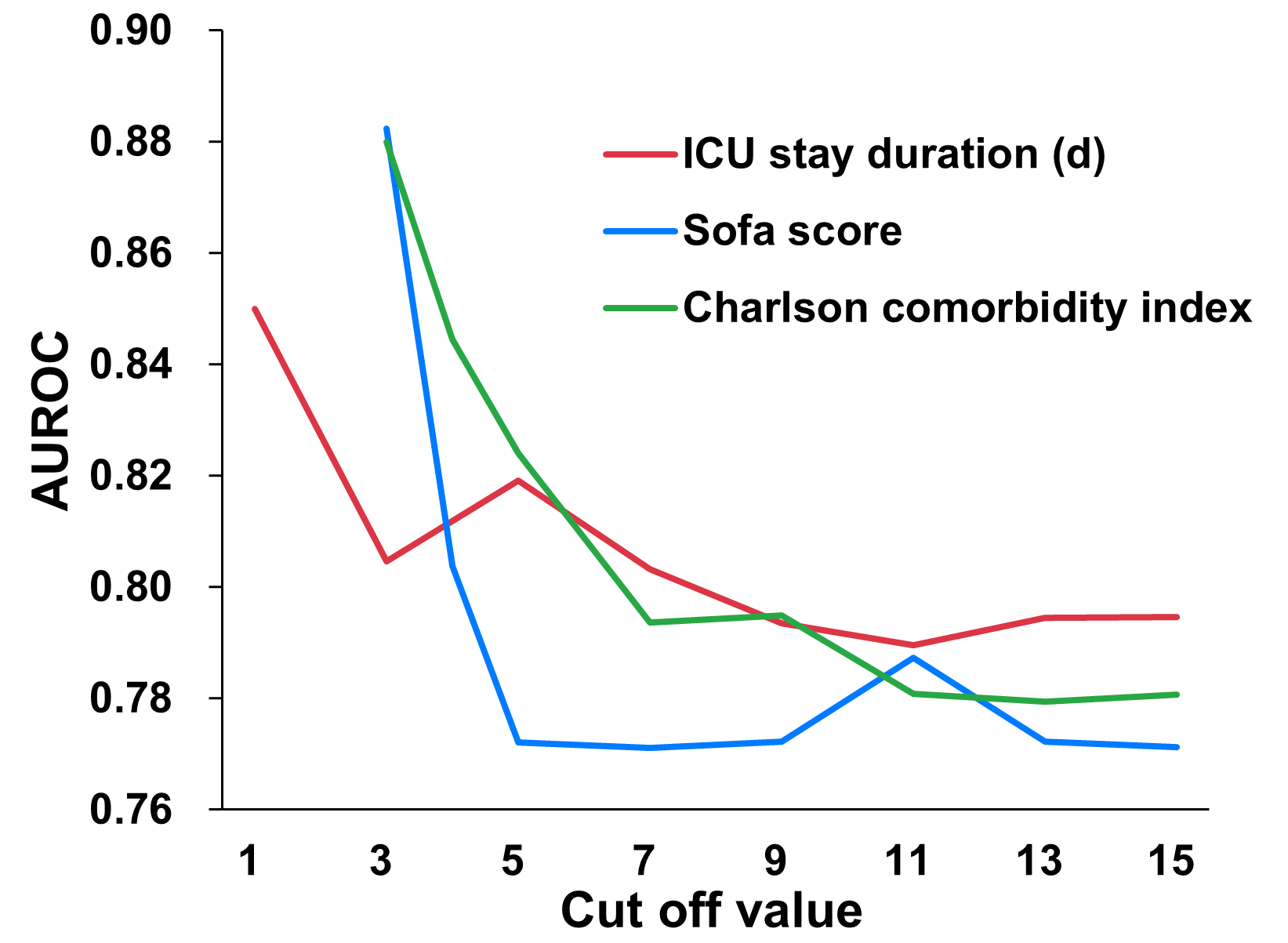
**

**Figure S5. Comparison of In-Hospital Mortality Between High-Risk and Low-Risk Populations Identified by the Model**


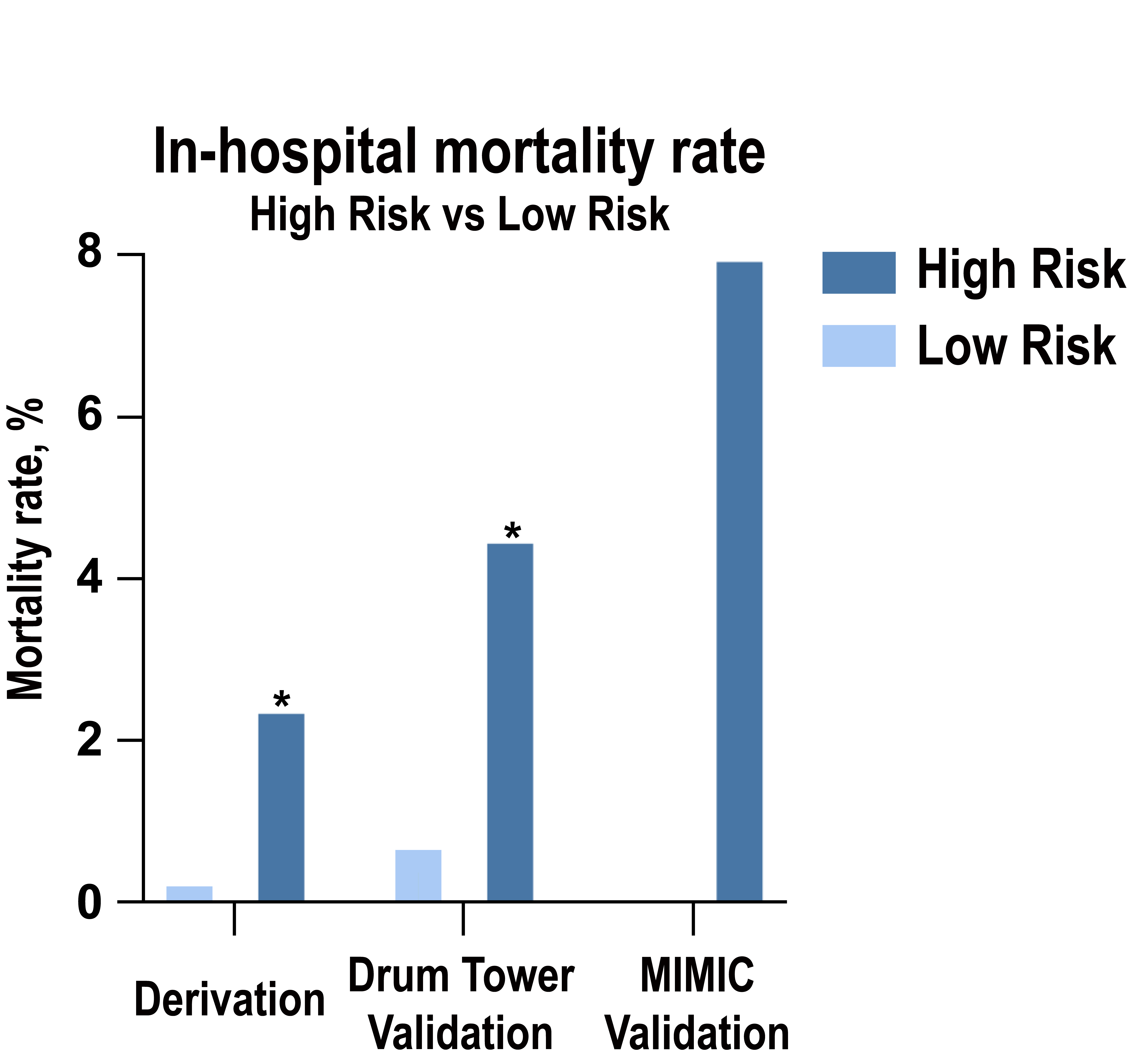


* This Figure shows the mortality rates (%) for high-risk and low-risk patients with gastrointestinal bleeding identified by the model across the Derivation, Drum Tower Validation, and MIMIC Validation cohorts. The high-risk group exhibits significantly higher mortality rates compared to the low-risk group, with p < .001. Statistical significance is indicated by an asterisk (*).

**Figure S6. Receiver Operating Characteristic Curves of the Model and the PRECISE-DAPT Score for Predicting In-hospital Mortality.**





**Table S1. The Calculation Process, Formula, and Results of Sample Size Determination.**

| **Method** | **Sample Size(n)** | **Formula** |
| --- | --- | --- |
| **Rule of Thumb** |  |  |
| 10 Events Per Candidate Predictor Parameter | 5667 | Each continuous candidate variable corresponds to one β coefficient. For categorical variables, the number of β coefficients is equal to the number of categories minus one. The required number of positive events is calculated as the total number of β coefficients summed and then multiplied by 10. |
| **Four-Step Procedure** |  |  |
| Sample Size for Precise Outcome Risk or Mean Estimates | 87 | 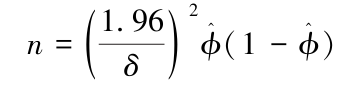 |
| Sample Size for Minimizing Prediction Error Across Individuals | 312 | 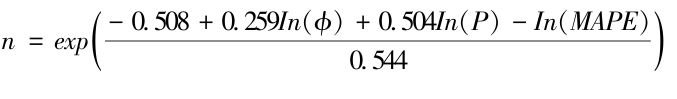 |
| Sample Size for Reducing Predictor Effect Shrinkage | 2391 | 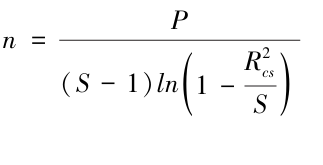 |
| Sample Size for Minimizing Optimism in Model Fit | 792 | 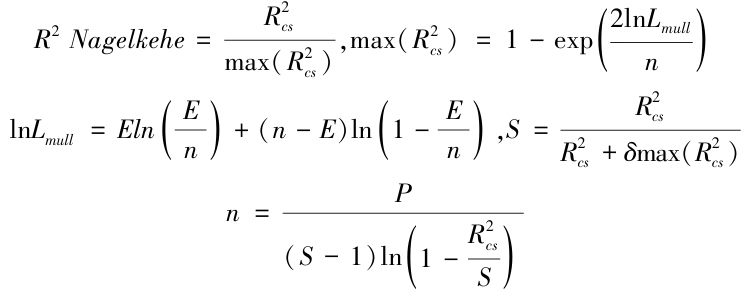 |

We identified a total of 34 candidate variables, with 15 predictive variables selected through LASSO. The expected proportion of the endpoint event was estimated to be 0.06. Based on these parameters, we calculated the sample size using the formula mentioned above.

For ease of application, we utilized and organized the pmsampsize package developed by Riley et al. in R to perform the calculations. The code has been uploaded to GitHub along with the other scripts used in this study. The parameters of the above formula are explained as follows:

ϕ: Expected proportion of the endpoint event

δ: Absolute error range

P: Number of predictive variables

MAPE: Mean absolute prediction error

S: Shrinkage factor


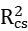
: Cox-Snell pseudo R-squared


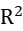
 Nagelkerke: Nagelkerke R-squared, an adjusted version of Cox-Snell R-squared

**Table S2. Admission Features for Machine Learning Models**

| **Features (n = 34)** | **Definition** |
| --- | --- |
| **Demographics** |  |
| Age | Age in years |
| Sex | Male or female |
| Body mass index | Weight in kg / height in m^2^ |
| Gastrointestinal bleeding history | Individual has previously experienced one or more episodes of gastrointestinal bleeding. |
| After percutaneous coronary intervention | Individual has previously undergone one or more percutaneous coronary interventions. |
| **Chronic comorbidities** | **Documented in the medical history of patients or meets the following diagnostic criteria** |
| Anemia | Hemoglobin concentration below 130 g/L for men and below 120 g/L for women, except in acute anemia. |
| Coagulation disorders | Chronic disorders caused by deficiencies or functional abnormalities of clotting factors, including hereditary conditions (such as hemophilia) and acquired cases, excluding coagulation dysfunctions that are caused by severe liver disease and those that occur acutely. |
| Hypertension | Recorded blood pressure >130/80 mmHg on three or more occasions. |
| Atrial fibrillation | Electrocardiogram showing no significant P waves and unequal R-R intervals owing to irregular ventricular responses. |
| Diabetes | Receiving anti-hyperglycemic medications including insulin. |
| Heart failure | Relying on information on history, physical examination, laboratory tests, cardiac imaging, and functional tests can confirm the diagnosis of a patient’s heart failure. |
| Cerebral vascular disease | A spectrum of diseases caused by cerebrovascular abnormalities primarily includes two major categories: ischemic (cerebral thrombosis and cerebral embolism) and hemorrhagic (cerebral hemorrhage). |
| Peripheral vascular disease | Chronic ischemic diseases of the limbs; common types include peripheral arterial disease, varicose veins, and deep vein thrombosis. |
| Chronic kidney disease | Impairment of kidney structure and function (with a history of more than 3 months) |
| Gastrointestinal ulcer | Chronic ulcers occurring in the stomach and duodenum. |

| **Features (n = 34)** | **Definition** |
| --- | --- |
| **Chronic comorbidities** | **Documented in history or meets the following diagnostic criteria.** |
| Gastritis | Various chronic inflammatory lesions of the gastric mucosa typically include chronic superficial gastritis, chronic erosive gastritis, chronic atrophic gastritis, etc. |
| Hyperlipidemia | Hypercholesterolemia (total cholesterol, TC ≥ 5.2 mmol/L), hypertriglyceridemia (triglycerides, TG ≥ 1.7 mmol/L), hyper-low-density lipoprotein cholesterolemia (low-density lipoprotein cholesterol, LDL-C ≥ 3.4 mmol/L), and low high-density lipoprotein cholesterolemia (high  LDL cholesterol, HDL-C <1.0 mmol/L) |
| Valvular disease | Diseases in which the four valves in the heart, i.e., the mitral, aortic, tricuspid, and pulmonary valves, develop functional or structural abnormalities. Valvular disease may result in valve stenosis or valve closure insufficiency. |
| **Admission examination** |  |
| White blood cell count, red blood cell count, platelets, hemoglobin, and hematocrit | Results from the patient’s first complete blood count after admission to the hospital. |
| Alanine aminotransferase, aspartate aminotransferase, bilirubin total, albumin, urea nitrogen, creatinine, and lactate dehydrogenase | Results from the patient’s first blood chemistry analysis after admission to the hospital. |
| Prothrombin time, activated partial thromboplastin time, and international normalized ratio | Results from the patient’s first coagulation test after admission to the hospital. |
| Ejection fraction | The ejection fraction from the patient’s first echocardiography after admission. |

* All listed chronic comorbidities exclude those that arose acutely or features that occurred after admission. Such features reflect the long-term condition of the patient’s body more accurately, are more relevant to postoperative gastrointestinal bleeding, and facilitate acquisition at the time of admission.

**Table S3. ICD-9/ICD-10 Codes are Used to Identify Chronic Comorbidities and Procedures**

| **Disease** | **ICD version** | **ICD code** |
| --- | --- | --- |
| Procedure：Coronary artery bypass grafting | 9 | '3610', '3611', '3612', '3613', '3614', '3615', '3616', '3617', '3619' |
|  | 10 | 'ICD codes that start with '021' and have long titles that include 'coronary artery' |
| Gastrointestinal bleeding | 9 | '53100', '53101', '53120', '53121', '53200', '53201', '53220', '53221', '53300', '53301', '53320', '53321', '53400', '53401', '53420', '53421', '53501', '53511', '53521', '53531', '53541', '53551', '53561', '53571', '5780', '5781', '5789' |
|  | 10 | 'K250', 'K252', 'K260', 'K262', 'K270', 'K272', 'K280', 'K282', 'K2901', 'K2961', 'K2971', 'K2981', 'K2991', 'K920', 'K921', 'K922' |
| Anemia | 9 | '2800','2801','2808','2809','2810','2811','2812','2813','2814','2818','2819','2820','2821','2822','2823','28240','28241','28242','28243','28244','28245','28246','28247','28249','2825','28260','28261','28262','28263','28264','28268','28269','2827','2828','2829','2830','28310','28311','28319','2832','2839','28401','28409','2841','28411','28412','28419','2842','28481','28489','2849','2850','2851','28521','28522','28529','2853','2858','2859' |
|  | 10 | 'D50','D500','D501','D508','D509','D51','D510','D511','D512','D513','D518','D519','D52','D520','D521','D528','D529','D53','D530','D531','D532','D538','D539','D55','E75242','D550','D551','D552','D553','D558','D559','D56','D560','D561','D562','D563','D564','D565','D568','D569','D57','D570','D5700','D5701','D5702','D571','D572','D5720','D5721','D57211','D57212','D57219','D573','D574','D5740','D5741','D57411','D57412','D57419','D578','D5780','D5781','D57811','D57812','D57819','D58','D580','D581','D582','D588','D589','D59','D590','D591','D592','D593','D594','D595','D596','D598','D599','D61','D610','D6101','D6109','D611','D612','D613','D618','D6181','D61810','D61811','D61818','D6182','D6189','D619','D61','D610','D6101','D6109','D611','D612','D613','D618','D6181','D61810','D61811','D61818','D6182','D6189','D619','D62','D63','D630','D631','D638','D64','D640','D641','D642','D643','D644','D648','D6481','D6489','D649' |
| Coagulation disorders | 9 | '2860','286','2862','2863','2864','2865','28652','28653','28659','2866','2867','2869' |
|  | 10 | 'D65','D66','D67','D68','D680','D681','D682','D683','D6831','D68311','D68312','D68318','D6832','D684','D685','D6851','D6852','D6859','D686','D6861','D6862','D6869','D688','D689','D69','D690','D691','D692','D693','D694','D6941','D6942','D6949','D695','D6951','D6959','D696','D698','D699' |

| **Disease** | **ICD version** | **ICD code** |
| --- | --- | --- |
| Hypertension | 9 | '4010','4011','4019' |
|  | 10 | 'I10' |
| Atrial fibrillation | 9 | '42731' |
|  | 10 | 'I48','I480','I481','I4811','I4819','I482','I4820','I4821','I483','I484','I489','I4891','I4892' |
| Diabetes | 9 | '2500','2501','2502','2503','2508','2509' |
|  | 10 | 'E100','E10l','E106','E108','E109','E110','E111','E116','E118','E119','E120','E121','E126','E128','E129','E130','E131','E136','E138','E139','E140', 'E141','E146','E148','E149' |
| Heart failure | 9 | '4281','42820','42821','42822','42823','42830','42831','42832','42833','42840','42841','42842','42843','4289','39891','40201','40211','40291','40401','40403','40411','40413','40491','40493','4254','4255','4257','4258','4259' |
|  | 10 | 'I43','I50','I099','I110','I130','I132','I255','I42','I426','I427','I428','I429','P290',' |
| Cerebral vascular disease | 9 | '430','431','4320','4321','4329','43300','43301','43310','43311','43320','43321','43330','43331','43380','43381','36234' |
|  | 10 | 'G45 to G459', 'G46 to G468', 'I60 to I609', 'I61 to I619', 'I62 to I629', 'I63 to I639', 'I65 to I659', 'I66 to I669', 'I67 to I679', 'I68 to I688', 'I69 to I699', 'H340 to H3403' |
| Peripheral vascular disease | 9 | '4400','4401','44020','44021','44022','44023','44024','44029','44030','44031','44032','4404','4408','4409','44100','44101','44102','44103','4411','4412','4413','4414','4415','4416','4417','4419','0930','4431','44321','44322','44323','44324','44329','44381','44382','44389','4439' |
|  | 10 | 'I70 to I7075', 'I7076 to I709', 'I71', 'I710 to I719', 'I731', 'I738', 'I7381', 'I7389', 'I739', 'I771', 'I790', 'K551', 'K558', 'K559', 'Z958', 'Z9581', 'Z95810 to Z95812', 'Z95818', 'Z9582', 'Z95820', 'Z95828', 'Z959' |
| Chronic kidney disease | 9 | '5820','5821','5822','5824','58281','58289','5829','5851','5852','5853','5854','5855','5856','5859','586','V560','V561','V562','V5631','V5632','V568','5830','5831','5832','5834','5836','5837','40301','40311','40391','40402','40403','40412','40413','40492','40493' |
|  | 10 | 'N18','N181','N182','N183','N184','N185','N186','N189','N19','I120','I131','I1310','I1311','N032','N033','N034','N035','N036','N037','N052','N053','N054','N055','N056','N057','N250','Z490','Z4901','Z4902','Z940','Z992' |
| Gastrointestinal ulcer | 9 | '53140','53141','53150','53151','53160','53161','53520','53170','53171','53240','53241','53250','53251','53260','53261','53270','5'3271','53340','53341','53350','53351','53360','53361','53370','53371','53440','53441','53450','53451','53460','53461','53470','53471' |
|  | 10 | 'K25','K254','K255','K256','K257','K26','K264','K265','K266','K267','K27','K274','K275','K276','K277','K28','L02225','K284','K285','K286','K287' |

| **Disease** | **ICD version** | **ICD code** |
| --- | --- | --- |
| Gastritis（Non acute） | 9 | '53501','53510','53511','53521','53530','53531','53540','53541','53550','53551','53560','53561','53570','53571' |
|  | 10 | 'K292','K2920','K2921','K293','K2930','K2931','K294','K2940','K2941','K295','K2950','K2951','K296','K2960','K2961','K297','K2970','K2971','K298','K2980','K2981','K299','K2990','K2991' |
| Hyperlipidemia | 9 | '2720','2721','2722','2723','2724' |
|  | 10 | 'E78','E780','E7800','E7801','E781','E782','E783','E784','E7841','E7849','E785' |
| Valvular disease | 9 | '3940','3941','3942','3949','3950','3951','3952','3959','3960','3961','3962','3963','3968','3969','3970','3971','3979','4240','4241','4242','4243' |
|  | 10 | 'I34','I340','I341','I342','I348','I349','I35','I350','I351','I352','I358','I359','I36','I360','I361','I362','I368','I369','I37','I370','I371','I372','I378','I379','I05','I050','I051','I052','I058','I059','I06','I060','I061','I062','I068','I069','I07','I070','I071','I072','I078','I079','I08','I080','I081','I082','I083','I088','I089' |

* The ICD_code for certain diseases is abbreviated as it has too many codes and takes up too much space.

Abbreviation: ICD-9, international classification of diseases, 9th revision; ICD-10, international classification of diseases, 10th revision.

**Table S4. Description of the Data Types and Missing Values for Each Feature**

| Features | Data Types | Missing Values | | |
| --- | --- | --- | --- | --- |
|  |  | Derivation Cohort | External Validation Cohort | |
|  |  | Multicenter | Drum Tower | MIMIC |
|  |  | (n=13399) | (n=2745) | (n=296) |
| **Demographics** |  |  |  |  |
| Age | Continuous | 0 (0) | 15 (0.55%) | 0 (0) |
| Sex | Categorical | 0 (0) | 68 (2.48%) | 0 (0) |
| Body mass index | Continuous | 454 (3.38%) | 61 (2.22%) | 18 (6.08%) |
| Gastrointestinal bleeding history | Categorical | 0 (0) | 0 (0) | 0 (0) |
| After percutaneous coronary intervention | Categorical | 0 (0) | 0 (0) | 0 (0) |
| **Chronic comorbidities** |  |  |  |  |
| Anemia | Categorical | 0 (0) | 0 (0) | 0 (0) |
| Coagulation disorders | Categorical | 0 (0) | 0 (0) | 0 (0) |
| Hypertension | Categorical | 0 (0) | 0 (0) | 0 (0) |
| Atrial fibrillation | Categorical | 0 (0) | 0 (0) | 0 (0) |
| Diabetes | Categorical | 0 (0) | 0 (0) | 0 (0) |
| Heart failure | Categorical | 0 (0) | 0 (0) | 0 (0) |
| Cerebral vascular disease | Categorical | 0 (0) | 0 (0) | 0 (0) |
| Peripheral vascular disease | Categorical | 0 (0) | 0 (0) | 0 (0) |
| Chronic kidney disease | Categorical | 0 (0) | 0 (0) | 0 (0) |
| Gastrointestinal ulcer | Categorical | 0 (0) | 0 (0) | 0 (0) |
| Gastritis | Categorical | 0 (0) | 0 (0) | 0 (0) |
| Hyperlipidemia | Categorical | 0 (0) | 0 (0) | 0 (0) |
| Valvular disease | Categorical | 0 (0) | 0 (0) | 0 (0) |
| **Admission examination** |  |  |  |  |
| White blood cells | Continuous | 1 (0.007%) | 51 (1.86%) | 0 (0) |
| Red blood cells | Continuous | 1 (0.007%) | 52 (1.89%) | 0 (0) |
| Platelets | Continuous | 2 (0.015%) | 53 (1.93%) | 0 (0) |
| Hemoglobin | Continuous | 22 (0.164%) | 53 (1.93%) | 0 (0) |
| Hematocrit | Continuous | 1 (0.007%) | 52 (1.89%) | 0 (0) |
| Alanine aminotransferase | Continuous | 2 (0.015%) | 88 (3.21%) | 38 (12.84%) |
| Aspartate aminotransferase | Continuous | 0 (0) | 61 (2.22%) | 37 (12.50%) |
| Total bilirubin | Continuous | 616 (4.59%) | 91 (3.32%) | 37 (12.50%) |
| Albumin | Continuous | 616 (4.59%) | 96 (3.50%) | 53 (17.90%) |
| Urea | Continuous | 160 (1.19%) | 59 (2.15%) | 0 (0) |
| Creatinine | Continuous | 1 (0.007%) | 59 (2.15%) | 0 (0) |
| Prothrombin time | Continuous | 16 (0.119%) | 83 (3.02%) | 0 (0) |
| Activated partial thromboplastin time | Continuous | 19 (0.142%) | 87 (3.17%) | 0 (0) |
| International normalized ratio | Continuous | 18 (0.134%) | 84 (3.06%) | 0 (0) |
| Lactate dehydrogenase | Continuous | 302 (2.25%) | 67 (2.44%) | 64 (21.62%) |
| Ejection fraction | Continuous | 866 (6.46%) | 230 (8.38%) | 39 (13.18%) |

**Table S5. Model Hyperparameter Settings, Ranges, and Rationale**

| **Model** | **Hyperparameters Settings and Ranges** | **Hyperparameter Rationale** |
| --- | --- | --- |
| **Linear** |  |  |
| *Logistic Regression* | C': [0.001, 0.01, 0.1, 1, 10, 100], 'penalty': ['l1', 'l2'] | C: Controls regularization strength. A smaller value increases regularization to prevent overfitting, while a larger value reduces it.  penalty: Determines regularization type: l1: Creates sparse models by setting some coefficients to zero. l2: Shrinks coefficients without eliminating features. |
| ***Non-linear, kernel-based methods*** |  |  |
| *Support Vector Machine* | C = np.logspace (-9, -2, 10, base=10) | C: Regularization parameter. Larger values reduce regularization and may cause overfitting, while smaller values simplify the model and prevent overfitting.  Kernel: Determines the decision boundary type. The RBF kernel is used for non-linear data, while the polynomial and sigmoid kernels are used for specific relationships. |
|  | 'kernel': ['rbf', 'poly','linear','sigmoid'] |  |
| ***Non-linear, ensemble learning methods*** |  |  |
| *Random Forest* | n_estimators = np.arange (100, 150, 10) | n_estimators: More trees improve performance but increase computation time. max_depth: Limits tree depth to control overfitting. min_samples_split: Controls how many samples are required to split a node. Higher values prevent overfitting. min_samples_leaf: Prevents the model from learning overly specific rules by requiring a minimum number of samples at leaf nodes. |
|  | max_depth = np.arange (2, 6, 1) |  |
|  | min_samples_split = np.arange (2, 5, 1) |  |
|  | min_samples_leaf = np.arange (3, 6, 1) |  |
| *EXtreme Gradient Boosting* | n_estimators':np.arange (8,15,1) | n_estimators: Number of boosting rounds or trees. More rounds can improve performance but also increase the risk of overfitting. max_depth: Determines the maximum depth of each tree, balancing complexity and overfitting. learning_rate: Controls the step size in boosting rounds. Smaller values often lead to better generalization. gamma: Regularization term, larger values make the model more conservative, thus reducing overfitting. min_child_weight: Minimum required sum of instance weights for a leaf node, used to prevent overfitting. |
|  | ,'max_depth':np.arange (1,6,1) |  |
|  | ,'learning_rate':np.arange (0.3,0.5,0.05) |  |
|  | ,'gamma':np.arange (0,0.03,0.01) |  |
|  | ,'min_child_weight':np.arange (0,5,1) |  |
| ***Probability*** |  |  |
| *Naive Bayes* | NA | Naive Bayes typically does not require hyperparameter tuning, as it makes strong independence assumptions between features. |
| ***Deep learning*** |  |  |
| *Multilayer Perceptron* | Hidden layers: 2 | Hidden layers: Determines the complexity of the model. More neurons allow for learning more complex patterns but may lead to overfitting. Configurations: A range of configurations is tested to balance between model complexity and performance. |
|  | Configurations: [ (64, 32), (32, 16), (16, 8)] |  |

**Table S6. Baseline Characteristics of Patients with and without Gastrointestinal Bleeding after Coronary Artery Bypass Grafting in the Derivation Cohort**

| **Variables** | **Derivation cohort (n=13399)** | |  |
| --- | --- | --- | --- |
|  | **No GIB (n=12596)** | **GIB (n=803)** | ***P* value** |
| Demographics |  |  |  |
| Age, median (IQR), years | 63 (12) | 66 (12) | ＜.001 |
| Sex, n (%) |  |  |  |
| Male | 9515 (75.54%) | 593 (73.85%) | ＜.001 |
| Female | 3081 (24.46%) | 210 (26.15%) |  |
| Body mass index, median (IQR) | 25.61 (4.07) | 25.23 (4.31) | ＜.001 |
| Gastrointestinal bleeding history, n (%) | 91 (0.72%) | 11 (1.37%) | .04 |
| After percutaneous coronary intervention, n (%) | 1467 (11.65%) | 111 (13.82%) | .06 |
| Chronic comorbidities, n (%) |  |  |  |
| Anemia | 1461 (11.6%) | 243 (30.26%) | ＜.001 |
| Coagulation disorders | 78 (0.62%) | 69 (8.59%) | ＜.001 |
| Hypertension | 5847 (46.42%) | 391 (48.69%) | .21 |
| Atrial fibrillation | 535 (4.25%) | 83 (10.34%) | ＜.001 |
| Diabetes | 4988 (39.6%) | 326 (40.6%) | .58 |
| Heart failure | 4629 (36.75%) | 485 (60.4%) | ＜.001 |
| Cerebral vascular disease | 1842 (14.62%) | 203 (25.28%) | ＜.001 |
| Peripheral vascular disease | 295 (2.34%) | 52 (6.48%) | ＜.001 |
| Chronic kidney disease | 427 (3.39%) | 187 (23.29%) | ＜.001 |
| Gastrointestinal ulcer | 158 (1.25%) | 51 (6.35%) | ＜.001 |
| Gastritis | 144 (1.14%) | 11 (1.37%) | .56 |
| Hyperlipidemia | 7940 (63.04%) | 473 (58.9%) | .02 |
| Valvular disease | 1840 (14.61%) | 247 (30.76%) | ＜.001 |
| Admission examination, median (IQR) |  |  |  |
| White blood cell count, ×10^9/L | 6.85 (2.79) | 7.43 (3.6) | ＜.001 |
| Red blood cell count, ×10^12/L | 4.42 (0.76) | 4.2 (1) | ＜.001 |
| Platelet count, ×10^9/L | 208 (77) | 185 (97) | ＜.001 |
| Hemoglobin, g/L | 137 (25) | 128 (33) | ＜.001 |
| Hematocrit, Proportion of 1.0 | 0.4 (0.07) | 0.38 (0.09) | ＜.001 |
| Alanine aminotransferase, µkat/L | 0.35 (0.3) | 0.32 (0.28) | .01 |
| Aspartate aminotransferase, µkat/L | 0.32 (0.17) | 0.33 (0.22) | ＜.001 |
| Bilirubin total, µmol/L | 11 (6.32) | 11.64 (7.55) | .001 |
| Albumin, g/L | 42.8 (4.8) | 41.3 (5.5) | ＜.001 |
| Urea, mmol/L | 5.74 (2.24) | 6.3 (3.44) | ＜.001 |
| Creatinine, μmol/L | 74.8 (21.1) | 81.2 (32.7) | ＜.001 |
| Prothrombin time, s | 11.4 (1) | 11.7 (1.5) | ＜.001 |

| **Variables** | **Derivation cohort (n=13399)** | |  |
| --- | --- | --- | --- |
|  | **No GIB (n=12596)** | **GIB (n=803)** | ***P* value** |
| Admission examination , median (IQR) |  |  |  |
| Activated partial thromboplastin time, s | 31 (4.3) | 31.6 (5.1) | ＜.001 |
| International normalized ratio | 1.01 (0.09) | 1.04 (0.13) | ＜.001 |
| Lactate dehydrogenase, µkat/L | 2.92 (0.75) | 3.14 (1.15) | ＜.001 |
| Ejection fraction, n (%) |  |  |  |
| ≥55% | 10150 (80.58%) | 461 (57.41%) | ＜.001 |
| 45-55% | 1610 (12.78%) | 162 (20.17%) |  |
| 30-45% | 808 (6.41%) | 160 (19.93%) |  |
| ＜30% | 28 (0.22%) | 20 (2.49%) |  |
| PRECISE-DAPT score, median (IQR) | 11.97 (10.93) | 19.46 (15.79) | ＜.001 |
| In-hospital mortality, n (%) | 59 (0.47%) | 64 (7.97%) | ＜.001 |

Abbreviations: IQR, interquartile range; PRECISE-DAPT score, Predicting Bleeding Complications in Patients Undergoing Stent Implantation and Subsequent Dual Antiplatelet Therapy.

**Table S7. Baseline Characteristics of Patients with and without Gastrointestinal Bleeding after Coronary Artery Bypass Grafting in the Drum Tower Validation Cohort**

| **Variables** | **Validation Cohort 1, Drum Tower (n=2745)** | |  |
| --- | --- | --- | --- |
|  | **No GIB (n=2566)** | **GIB (n=179)** | ***P* value** |
| Demographics |  |  |  |
| Age, median (IQR), years | 70 (14） | 73 (14） | .001 |
| Sex, n (%) |  |  |  |
| Male | 1781 (69.41%) | 140 (78.21%) | .01 |
| Female | 785 (30.59%) | 39 (21.79%) |  |
| Body mass index, median (IQR) | 24.4 (4.3) | 24.5 (4.5) | .85 |
| Gastrointestinal bleeding history, n (%) | 12 (0.47%) | 17 (9.5%) | ＜.001 |
| After percutaneous coronary intervention, n (%) | 234 (9.12%) | 28 (15.64%) | .004 |
| Chronic comorbidities, n (%) |  |  |  |
| Anemia | 272 (10.6%) | 47 (26.26%) | ＜.001 |
| Coagulation disorders | 7 (0.27%) | 10 (5.59%) | ＜.001 |
| Hypertension | 1559 (60.76%) | 132 (73.74%) | .001 |
| Atrial fibrillation | 233 (9.08%) | 25 (13.97%) | .03 |
| Diabetes | 768 (29.93%) | 64 (35.75%) | .1 |
| Heart failure | 1018 (39.67%) | 96 (53.63%) | ＜.001 |
| Cerebral vascular disease | 434 (16.91%) | 50 (27.93%) | ＜.001 |
| Peripheral vascular disease | 169 (6.59%) | 27 (15.08%) | ＜.001 |
| Chronic kidney disease | 98 (3.82%) | 35 (19.55%) | ＜.001 |
| Gastrointestinal ulcer | 23 (0.9%) | 14 (7.82%) | ＜.001 |
| Gastritis | 80 (3.12%) | 5 (2.79%) | .81 |
| Hyperlipidemia | 1586 (61.8%) | 103 (57.54%) | .26 |
| Valvular disease | 388 (14.26%) | 67 (37.43%) | ＜.001 |
| Admission examination, median (IQR) |  |  |  |
| White blood cell count, ×10^9/L | 6.2 (2.3) | 7.2 (4.2) | ＜.001 |
| Red blood cell count, ×10^12/L | 4.4 (0.71) | 4.2 (0.81) | ＜.001 |
| Platelet count, ×10^9/L | 193 (73) | 175 (88) | ＜.001 |
| Hemoglobin, g/L | 134 (22) | 127 (30) | ＜.001 |
| Hematocrit, Proportion of 1.0 | 0.399 (0.06) | 0.379 (0.078) | ＜.001 |
| Alanine aminotransferase, µkat/L | 0.36 (0.31) | 0.35 (0.42) | .78 |
| Aspartate aminotransferase, µkat/L | 0.37 (0.23) | 0.48 (0.36) | ＜.001 |
| Bilirubin total, µmol/L | 10.8 (6.7) | 11.5 (8.8) | .02 |
| Albumin, g/L | 39.8 (4) | 37.5 (4) | ＜.001 |
| Urea, mmol/L | 6.1 (2.6) | 7.2 (4.6) | ＜.001 |
| Creatinine, μmol/L | 70.6 (24) | 84.2 (33.5) | ＜.001 |
| Prothrombin time, s | 11.4 (1.2) | 12.2 (1.5) | ＜.001 |

| **Variables** | **Validation Cohort 1, Drum Tower (n=2745)** | |  |
| --- | --- | --- | --- |
|  | **No GIB (n=2566)** | **GIB (n=179)** | ***P* value** |
| Admission examination , median (IQR) |  |  |  |
| Activated partial thromboplastin time, s | 27.7 (4.1) | 28.5 (4.8) | .001 |
| International normalized ratio | 1 (0.11) | 1.06 (0.13) | ＜.001 |
| Lactate dehydrogenase, µkat/L | 3.31 (1.35) | 4.66 (4.93) | ＜.001 |
| Ejection fraction, n (%) |  |  |  |
| ≥55% | 1517 (59.12%) | 55 (30.73%) | ＜.001 |
| 45-55% | 547 (21.32%) | 32 (17.88%) |  |
| 30-45% | 462 (18%) | 81 (45.25%) |  |
| ＜30% | 40 (1.56%) | 11 (6.15%) |  |
| PRECISE-DAPT score, median (IQR) | 14.2 (10.3) | 22.83 (17.18) | ＜.001 |
| In-hospital mortality, n (%) | 41 (1.59%) | 28 (15.64%) | ＜.001 |

Abbreviations: IQR, interquaqrtile range; PRECISE-DAPT score, Predicting Bleeding Complications in Patients Undergoing Stent Implantation and Subsequent Dual Antiplatelet Therapy.

**Table S8. Baseline Characteristics of Patients with and without Gastrointestinal Bleeding after Coronary Artery Bypass Grafting in the MIMIC Validation Cohort**

| **Variables** | **Validation Cohort 2, MIMIC (n=296)** | |  |
| --- | --- | --- | --- |
|  | **No GIB (n=120)** | **GIB (n=176)** | ***P* value** |
| Demographics |  |  |  |
| Age, median (IQR), years | 72.3 (15.14） | 75.1 (13.87） | .21 |
| Sex, n (%) |  |  |  |
| Male | 76 (63.33%) | 122 (69.32%) | .28 |
| Female | 44 (36.67%) | 54 (30.68%) |  |
| Body mass index, median (IQR) | 30.2 (7.01) | 29.74 (7.04) | .23 |
| Gastrointestinal bleeding history, n (%) | 10 (8.33%) | 20 (11.36%) | .37 |
| After percutaneous coronary intervention, n (%) | 13 (10.83%) | 15 (8.52%) | .51 |
| Chronic comorbidities, n (%) |  |  |  |
| Anemia | 40 (33.33%) | 82 (46.59%) | .02 |
| Coagulation disorders | 14 (11.67%) | 78 (44.32%) | ＜.001 |
| Hypertension | 61 (50.83%) | 70 (39.77%) | .06 |
| Atrial fibrillation | 56 (46.67%) | 129 (73.3%) | ＜.001 |
| Diabetes | 44 (36.67%) | 56 (31.82%) | .39 |
| Heart failure | 44 (36.67%) | 102 (57.95%) | ＜.001 |
| Cerebral vascular disease | 17 (14.17%) | 36 (20.45%) | .17 |
| Peripheral vascular disease | 23 (19.17%) | 48 (27.27%) | .11 |
| Chronic kidney disease | 31 (25.83%) | 82 (46.59%) | ＜.001 |
| Gastrointestinal ulcer | 1 (0.83%) | 10 (5.68%) | .03 |
| Gastritis | 6 (5%) | 4 (2.27%) | .2 |
| Hyperlipidemia | 88 (73.33%) | 113 (64.2%) | .1 |
| Valvular disease | 35 (29.17%) | 72 (40.91%) | .04 |
| Admission examination, median (IQR) |  |  |  |
| White blood cell count, ×10^9/L | 10.45 (6.5) | 11.9 (8) | .001 |
| Red blood cell count, ×10^12/L | 3.3 (0.77) | 3.2 (0.8) | .06 |
| Platelet count, ×10^9/L | 191.5 (143) | 166 (126) | .03 |
| Hemoglobin, g/L | 101.5 (27) | 96 (21) | .02 |
| Hematocrit, Proportion of 1.0 | 0.299 (0.07) | 0.292 (0.066) | .12 |
| Alanine aminotransferase, µkat/L | 0.42 (0.52) | 0.39 (0.63) | .78 |
| Aspartate aminotransferase, µkat/L | 0.59 (0.75) | 0.63 (1.17) | .06 |
| Bilirubin total, µmol/L | 10.26 (8.55) | 11.97 (14.96) | .08 |
| Albumin, g/L | 34.15 (5.94) | 32.33 (6.1) | .01 |
| Urea, mmol/L | 8.211 (5.71) | 10.71 (10.26) | .001 |
| Creatinine, μmol/L | 97.24 (53.04) | 123.76 (106.08) | ＜.001 |
| Prothrombin time, s | 13.6 (3.7) | 15 (5) | ＜.001 |

| **Variables** | **Validation Cohort 2, MIMIC (n=296)** | |  |
| --- | --- | --- | --- |
|  | **No GIB (n=120)** | **GIB (n=176)** | ***P* value** |
| Admission examination , median (IQR) |  |  |  |
| Activated partial thromboplastin time, s | 31.05 (10.5) | 32.8 (15.9) | .04 |
| International normalized ratio | 1.2 (0.4) | 1.4 (0.5) | ＜.001 |
| Lactate dehydrogenase, µkat/L | 4.38 (2.87) | 5.58 (3.22) | ＜.001 |
| Ejection fraction, n (%) |  |  |  |
| ≥55 | 62 (51.67) | 47 (26.7) | ＜.001 |
| 45-55 | 33 (27.5) | 43 (24.43) |  |
| 30-45 | 18 (15) | 54 (30.68) |  |
| ＜30 | 7 (5.83) | 32 (18.18) |  |
| PRECISE-DAPT score, median (IQR) | 35.58 (20.39) | 43.87 (19.05) | ＜.001 |
| In-hospital mortality, n (%) | 3 (2.5%) | 19 (10.8%) | ＜.001 |

Abbreviations: IQR, interquartile range; PRECISE-DAPT score, Predicting Bleeding Complications in Patients Undergoing Stent Implantation and Subsequent Dual Antiplatelet Therapy.

**Table S9. Results of Five Feature Selection Methods**

| **Features** | **LASSO** | **K-best** | **MI** | **RFE** | **COM** |
| --- | --- | --- | --- | --- | --- |
| Age | √ |  |  |  |  |
| Anemia | √ | √ | √ |  | √ |
| Coagulation disorders | √ | √ | √ | √ | √ |
| Heart failure | √ | √ | √ |  | √ |
| Cerebral vascular disease | √ |  |  |  |  |
| Chronic kidney disease | √ | √ | √ | √ | √ |
| Gastrointestinal ulcer | √ |  |  |  |  |
| Valvular disease | √ | √ | √ |  | √ |
| White blood cell count | √ | √ |  | √ | √ |
| Red blood cell count |  | √ | √ | √ | √ |
| Platelet count | √ | √ | √ | √ | √ |
| Hemoglobin | √ | √ | √ |  | √ |
| Aspartate aminotransferase |  | √ |  | √ |  |
| Bilirubin total |  |  |  | √ |  |
| Albumin | √ | √ | √ | √ | √ |
| Urea |  | √ | √ | √ | √ |
| Creatinine |  | √ | √ | √ | √ |
| Prothrombin time |  |  | √ | √ |  |
| Activated partial thromboplastin time |  |  |  | √ |  |
| International normalized ratio | √ | √ | √ | √ | √ |
| Lactate dehydrogenase | √ | √ | √ | √ | √ |
| Ejection fraction | √ | √ | √ | √ | √ |

Abbreviations: LASSO, least absolute shrinkage and selection operator; K-Best, k-best feature selection; MI, mutual information; RFE, recursive feature elimination; COM, combined method, including features appearing more than three times in the first four feature selection methods

**Table S10. Optimal Hyperparameters for Each Model Configuration**

| **Model** | **Hyperparameters** |
| --- | --- |
| LASSO |  |
| *Logistic Regression* | C: 10, penalty: l1 |
| *Support Vector Machine* | C: 0.00001, kernel: linear |
| *Random Forest* | max_depth: 5, min_samples_leaf: 4, min_samples_split: 2, n_estimators: 140 |
| *EXtreme Gradient Boosting* | n_estimators: 27, max_depth: 2, learning_rate: 0.32, gamma: 0.0, min_child_weight: 0.2 |
| *Multilayer Perceptron* | Hidden layers: 2, Configurations: (32, 16) |
| *K-Best* |  |
| *Logistic Regression* | C: 10, penalty: l1 |
| *Support Vector Machine* | C: 0.0001, kernel: linear |
| *Random Forest* | max_depth: 5, min_samples_leaf: 4, min_samples_split: 2, n_estimators: 130 |
| *EXtreme Gradient Boosting* | gamma: 0.0, learning_rate: 0.35, max_depth: 3, min_child_weight: 0, n_estimators: 11 |
| *Multilayer Perceptron* | Hidden layers: 2, Configurations: (32, 16) |
| Mutual Information |  |
| *Logistic Regression* | C: 1, penalty: l2 |
| *Support Vector Machine* | C: 0.0001, kernel: linear |
| *Random Forest* | max_depth: 5, min_samples_leaf: 4, min_samples_split: 2, n_estimators: 140 |
| *EXtreme Gradient Boosting* | gamma: 0.0, learning_rate: 0.35, max_depth: 4, min_child_weight: 2, n_estimators: 8 |
| *Multilayer Perceptron* | Hidden layers: 2, Configurations : (32, 16) |
| Recursive Feature Elimination |  |
| *Logistic Regression* | C: 10, penalty: l2 |
| *Support Vector Machine* | C: 0.0001, kernel: linear |
| *Random Forest* | max_depth: 3, min_samples_leaf: 3, min_samples_split: 2, n_estimators: 140 |
| *EXtreme Gradient Boosting* | gamma: 0.0, learning_rate: 0.4, max_depth: 2, min_child_weight: 3, n_estimators: 12 |
| *Multilayer Perceptron* | Hidden layers: 2, Configurations: (16, 8) |
| Combined Method |  |
| *Logistic Regression* | C: 10, penalty: l1 |
| *Support Vector Machine* | C: 0.00001, kernel: linear |
| *Random Forest* | max_depth: 5, min_samples_leaf: 5, min_samples_split: 2, n_estimators: 120 |
| *EXtreme Gradient Boosting* | gamma: 0.0, learning_rate: 0.3, max_depth: 3, min_child_weight: 0, n_estimators: 10 |
| *Multilayer Perceptron* | Hidden layers: 2, Configurations: (32, 16) |

**Table S11. Average AUROC and Its 95% Confidence Interval for Each Model in the Training Set (Comprising Four Parts of the Data from the Five-fold Cross-validation Conducted on the Derivation Cohort)**

| **Model** | **Mean** | **95% Confidence Interval** |
| --- | --- | --- |
| LASSO |  |  |
| *Logistic Regression* | 0.8289 | 0.8257-0.8322 |
| *Support Vector Machine* | 0.7978 | 0.7814-0.8142 |
| *Random Forest* | 0.8453 | 0.8425-0.8482 |
| *Naive Bayes* | 0.8123 | 0.8091-0.8155 |
| *EXtreme Gradient Boosting* | 0.8538 | 0.8504-0.8571 |
| *Multilayer Perceptron* | 0.8239 | 0.8229-0.8249 |
| *K-Best* |  |  |
| *Logistic Regression* | 0.8143 | 0.8112-0.8174 |
| *Support Vector Machine* | 0.7835 | 0.7713-0.7957 |
| *Random Forest* | 0.8336 | 0.8302-0.837 |
| *Naive Bayes* | 0.7960 | 0.7928-0.7992 |
| *EXtreme Gradient Boosting* | 0.8419 | 0.839-0.8449 |
| *Multilayer Perceptron* | 0.8061 | 0.8051-0.8071 |
| Mutual Information |  |  |
| *Logistic Regression* | 0.8107 | 0.8076-0.8139 |
| *Support Vector Machine* | 0.7735 | 0.7502-0.7968 |
| *Random Forest* | 0.8334 | 0.8302-0.8367 |
| *Naive Bayes* | 0.8034 | 0.7999-0.8069 |
| *EXtreme Gradient Boosting* | 0.8473 | 0.8444-0.8503 |
| *Multilayer Perceptron* | 0.8002 | 0.7992-0.8012 |
| Recursive Feature Elimination |  |  |
| *Logistic Regression* | 0.7806 | 0.7768-0.7843 |
| *Support Vector Machine* | 0.7380 | 0.7042-0.7717 |
| *Random Forest* | 0.7948 | 0.7906-0.799 |
| *Naive Bayes* | 0.7625 | 0.7579-0.7671 |
| *EXtreme Gradient Boosting* | 0.8049 | 0.8011-0.8088 |
| *Multilayer Perceptron* | 0.7779 | 0.7767-0.7791 |
| Combined Method |  |  |
| *Logistic Regression* | 0.8145 | 0.8114-0.8176 |
| *Support Vector Machine* | 0.7800 | 0.7697-0.7902 |
| *Random Forest* | 0.8360 | 0.8326-0.8394 |
| *Naive Bayes* | 0.8016 | 0.7982-0.8049 |
| *EXtreme Gradient Boosting* | 0.8376 | 0.834-0.8412 |
| *Multilayer Perceptron* | 0.8053 | 0.8043-0.8063 |

**Table S12. Average AUROC and Its 95% Confidence Interval for Each Model in the Internal Validation Set (Comprising the Remaining One Part of Data from the Five-fold Cross-validation)**

| **Model** | **Mean** | **95% Confidence Interval** |
| --- | --- | --- |
| LASSO |  |  |
| *Logistic Regression* | 0.8244 | 0.8124-0.8385 |
| *Support Vector Machine* | 0.7977 | 0.7799-0.8154 |
| *Random Forest* | 0.8208 | 0.8077-0.834 |
| *Naive Bayes* | 0.8111 | 0.7978-0.8244 |
| *EXtreme Gradient Boosting* | 0.8265 | 0.814-0.839 |
| *Multilayer Perceptron* | 0.8235 | 0.8194-0.8276 |
| *K-Best* |  |  |
| *Logistic Regression* | 0.8109 | 0.7985-0.8232 |
| *Support Vector Machine* | 0.7820 | 0.7648-0.7992 |
| *Random Forest* | 0.8084 | 0.7942-0.8227 |
| *Naive Bayes* | 0.7953 | 0.7828-0.8079 |
| *EXtreme Gradient Boosting* | 0.8078 | 0.7945-0.8211 |
| *Multilayer Perceptron* | 0.8058 | 0.8019-0.8098 |
| Mutual Information |  |  |
| *Logistic Regression* | 0.8084 | 0.7958-0.8209 |
| *Support Vector Machine* | 0.7700 | 0.7403-0.7998 |
| *Random Forest* | 0.8088 | 0.7945-0.823 |
| *Naive Bayes* | 0.8018 | 0.789-0.8146 |
| *EXtreme Gradient Boosting* | 0.8035 | 0.7893-0.8177 |
| *Multilayer Perceptron* | 0.8000 | 0.796-0.804 |
| Recursive Feature Elimination |  |  |
| *Logistic Regression* | 0.7776 | 0.7625-0.7926 |
| *Support Vector Machine* | 0.7365 | 0.7008-0.7723 |
| *Random Forest* | 0.7775 | 0.7602-0.7948 |
| *Naive Bayes* | 0.7609 | 0.7449-0.7769 |
| *EXtreme Gradient Boosting* | 0.7773 | 0.7619-0.7928 |
| *Multilayer Perceptron* | 0.7776 | 0.7729-0.7823 |
| Combined Method |  |  |
| *Logistic Regression* | 0.8112 | 0.7989-0.8236 |
| *Support Vector Machine* | 0.7797 | 0.7628-0.7967 |
| *Random Forest* | 0.8106 | 0.7972-0.8241 |
| *Naive Bayes* | 0.8009 | 0.7875-0.8142 |
| *EXtreme Gradient Boosting* | 0.8079 | 0.7945-0.8213 |
| *Multilayer Perceptron* | 0.8050 | 0.8011-0.809 |

**Table S13. Mean Value of Brier Scores and Its 95% Confidence Interval for Each Model in the Training Set**

| **Model** | **Mean** | **95% Confidence Interval** |
| --- | --- | --- |
| LASSO |  |  |
| *Logistic Regression* | 0.0461 | 0.0459-0.0463 |
| *Support Vector Machine* | 0.0715 | 0.0668-0.0763 |
| *Random Forest* | 0.0454 | 0.0451-0.0456 |
| *Naive Bayes* | 0.0944 | 0.0941-0.0946 |
| *EXtreme Gradient Boosting* | 0.0433 | 0.0431-0.0435 |
| *Multilayer Perceptron* | 0.0479 | 0.0475-0.0483 |
| *K-Best* |  |  |
| *Logistic Regression* | 0.0473 | 0.047-0.0475 |
| *Support Vector Machine* | 0.0559 | 0.0549-0.0569 |
| *Random Forest* | 0.0448 | 0.0446-0.045 |
| *Naive Bayes* | 0.0856 | 0.0849-0.0863 |
| *EXtreme Gradient Boosting* | 0.0435 | 0.0433-0.0437 |
| *Multilayer Perceptron* | 0.0492 | 0.0488-0.0496 |
| Mutual Information |  |  |
| *Logistic Regression* | 0.0480 | 0.0478-0.0482 |
| *Support Vector Machine* | 0.0584 | 0.0567-0.0601 |
| *Random Forest* | 0.0452 | 0.045-0.0455 |
| *Naive Bayes* | 0.0849 | 0.0843-0.0856 |
| *EXtreme Gradient Boosting* | 0.0425 | 0.0423-0.0427 |
| *Multilayer Perceptron* | 0.0495 | 0.0491-0.0499 |
| Recursive Feature Elimination |  |  |
| *Logistic Regression* | 0.0486 | 0.0483-0.0488 |
| *Support Vector Machine* | 0.0595 | 0.0583-0.0606 |
| *Random Forest* | 0.0491 | 0.0489-0.0494 |
| *Naive Bayes* | 0.0859 | 0.0851-0.0866 |
| *EXtreme Gradient Boosting* | 0.0464 | 0.0462-0.0467 |
| *Multilayer Perceptron* | 0.0503 | 0.0499-0.0506 |
| Combined Method |  |  |
| *Logistic Regression* | 0.0473 | 0.0471-0.0476 |
| *Support Vector Machine* | 0.0635 | 0.0615-0.0656 |
| *Random Forest* | 0.0453 | 0.0451-0.0455 |
| *Naive Bayes* | 0.0855 | 0.0848-0.0862 |
| *EXtreme Gradient Boosting* | 0.0444 | 0.0441-0.0446 |
| *Multilayer Perceptron* | 0.0493 | 0.0489-0.0497 |

**Table S14. Mean Value of Brier Scores and Its 95% Confidence Interval for Each Model in the Internal Validation Set**

| **Model** | **Mean** | **95% Confidence Interval** |
| --- | --- | --- |
| LASSO |  |  |
| *Logistic Regression* | 0.0465 | 0.0457-0.0474 |
| *Support Vector Machine* | 0.0719 | 0.0673-0.0765 |
| *Random Forest* | 0.0481 | 0.0472-0.0491 |
| *Naive Bayes* | 0.0946 | 0.0934-0.0957 |
| *EXtreme Gradient Boosting* | 0.0463 | 0.0454-0.0472 |
| *Multilayer Perceptron* | 0.0483 | 0.0473-0.0493 |
| *K-Best* |  |  |
| *Logistic Regression* | 0.0478 | 0.0469-0.0486 |
| *Support Vector Machine* | 0.0565 | 0.0554-0.0576 |
| *Random Forest* | 0.0481 | 0.0472-0.049 |
| *Naive Bayes* | 0.0859 | 0.0844-0.0874 |
| *EXtreme Gradient Boosting* | 0.0475 | 0.0466-0.0484 |
| *Multilayer Perceptron* | 0.0493 | 0.0483-0.0503 |
| Mutual Information |  |  |
| *Logistic Regression* | 0.0483 | 0.0474-0.0493 |
| *Support Vector Machine* | 0.0589 | 0.0572-0.0606 |
| *Random Forest* | 0.0483 | 0.0474-0.0492 |
| *Naive Bayes* | 0.0854 | 0.0841-0.0867 |
| *EXtreme Gradient Boosting* | 0.0480 | 0.0471-0.0488 |
| *Multilayer Perceptron* | 0.0497 | 0.0487-0.0507 |
| Recursive Feature Elimination |  |  |
| *Logistic Regression* | 0.0491 | 0.0481-0.05 |
| *Support Vector Machine* | 0.0600 | 0.0587-0.0613 |
| *Random Forest* | 0.0503 | 0.0493-0.0514 |
| *Naive Bayes* | 0.0866 | 0.085-0.0882 |
| *EXtreme Gradient Boosting* | 0.0487 | 0.0478-0.0496 |
| *Multilayer Perceptron* | 0.0504 | 0.0494-0.0514 |
| Combined Method |  |  |
| *Logistic Regression* | 0.0478 | 0.0469-0.0487 |
| *Support Vector Machine* | 0.0639 | 0.0619-0.0659 |
| *Random Forest* | 0.0483 | 0.0473-0.0492 |
| *Naive Bayes* | 0.0858 | 0.0844-0.0872 |
| *EXtreme Gradient Boosting* | 0.0477 | 0.0468-0.0486 |
| *Multilayer Perceptron* | 0.0494 | 0.0485-0.0504 |

**Table S15. Univariate and Multivariate Analysis of Risk Factors for Mortality in Patients Across All Cohorts**

| Variables | Univariate analysis | | Multivariate analysis | |
| --- | --- | --- | --- | --- |
|  | OR (95%CI) | ***P* value** | OR (95%CI) | ***P* value** |
| Demographics |  |  |  |  |
| Age (S.D.) | 1.947 (1.658-2.286) | ＜.001 | 1.09 (0.895-1.327) | .39 |
| Sex | 0.682 (0.568-0.819) | ＜.001 | 0.797 (0.641-0.992) | **.04** |
| Body mass index (S.D.) | 0.932 (0.803-1.081) | .35 |  |  |
| Gastrointestinal bleeding history | 5.807 (3.1-10.879) | ＜.001 | 0.385 (0.138-1.072) | .07 |
| After percutaneous coronary intervention | 1.128 (0.751-1.694) | .56 |  |  |
| Chronic comorbidities |  |  |  |  |
| Anemia | 4.063 (3.053-5.407) | ＜.001 | 1.498 (1.052-2.133) | **.03** |
| Coagulation disorders | 21.568 (15.18-30.644) | ＜.001 | 2.94 (1.772-4.878) | **＜.001** |
| Hypertension | 1.412 (1.075-1.854) | .01 | 1.263 (0.937-1.704) | .13 |
| Atrial fibrillation | 4.342 (3.13-6.025) | ＜.001 | 0.696 (0.454-1.067) | .09 |
| Diabetes | 1.142 (0.868-1.502) | .34 |  |  |
| Heart failure | 2.575 (1.951-3.398) | ＜.001 | 1.28 (0.934-1.755) | .12 |
| Cerebral vascular disease | 2.771 (2.08-3.691) | ＜.001 | 1.938 (1.404-2.674) | **＜.001** |
| Peripheral vascular disease | 4.909 (3.357-7.18) | ＜.001 | 1.245 (0.795-1.95) | .34 |
| Chronic kidney disease | 9.934 (7.415-13.308) | ＜.001 | 1.829 (1.149-2.91) | **.011** |
| Gastrointestinal ulcer | 5.748 (3.446-9.587) | ＜.001 | 2.113 (1.117-3.998) | **.021** |
| Gastritis | 2.565 (1.252-5.257) | .01 | 1.125 (0.486-2.603) | .78 |
| Hyperlipidemia | 0.818 (0.623-1.076) | .15 |  |  |
| Valvular disease | 2.974 (2.257-3.917) | ＜.001 | 1.265 (0.915-1.747) | .15 |
| Admission examination (S.D.) |  |  |  |  |
| White blood cell count | 1.353 (1.152-1.589) | ＜.001 | 0.764 (0.626-0.934) | **.008** |
| Red blood cell count | 0.638 (0.549-0.741) | ＜.001 | 1.257 (0.906-1.743) | .17 |
| Platelet count | 0.626 (0.534-0.733) | ＜.001 | 0.9 (0.763-1.062) | .21 |
| Hemoglobin | 0.598 (0.514-0.695) | ＜.001 | 0.939 (0.64-1.376) | .75 |
| Hematocrit | 0.634 (0.546-0.736) | ＜.001 | 1.208 (0.846-1.725) | .30 |
| Alanine aminotransferase | 1.231 (1.004-1.51) | .046 | 0.996 (0.754-1.316) | .98 |
| Aspartate aminotransferase | 2.342 (1.937-2.833) | ＜.001 | 1.302 (0.945-1.792) | .11 |
| Bilirubin total | 1.538 (1.254-1.886) | ＜.001 | 1.16 (0.922-1.46) | .20 |
| Albumin | 0.447 (0.378-0.527) | ＜.001 | 0.875 (0.723-1.059) | .17 |
| Urea | 2.81 (2.312-3.415) | ＜.001 | 1.36 (1.045-1.771) | **.02** |
| Creatinine | 2.299 (1.874-2.821) | ＜.001 | 0.553 (0.403-0.758) | **＜.001** |
| Prothrombin time | 2.795 (2.284-3.421) | ＜.001 | 1.146 (0.723-1.816) | .56 |
| Activated partial thromboplastin time | 1.739 (1.405-2.152) | ＜.001 | 1.243 (0.997-1.551) | .05 |
| International normalized ratio | 2.569 (2.139-3.086) | ＜.001 | 0.865 (0.56-1.336) | .51 |
| Lactate dehydrogenase | 2.667 (2.246-3.168) | ＜.001 | 0.99 (0.769-1.275) | .94 |
| Ejection fraction drop value (per 5%) | 2.134 (1.848-2.465) | ＜.001 | 1.186 (0.982-1.431) | .08 |
| **PRECISE-DAPT score** | 1.071 (1.062-1.08) | ＜.001 | 1.046 (1.021-1.072) | **＜.001** |
| **Risk probability (%)** | 1.056 (1.051-1.06) | ＜.001 | 1.017 (1.004-1.031) | **.009** |
| **Risk level** | 12.864 (8.467-19.546) | ＜.001 | 2.98 (1.784-4.978) | **＜.001** |

**Table S16. Univariate analysis of preoperative medications in model-differentiated high- and low-risk populations**

| **Preoperative medications** | **High risk** | | |  | **Low risk** | | |
| --- | --- | --- | --- | --- | --- | --- | --- |
|  | **No GIB** | **GIB** | ***P* value** |  | **No GIB** | **GIB** | ***P* value** |
| **All cohorts** | n=5165 | n=1001 |  |  | n=10117 | n=157 |  |
| Proton pump Inhibitors, n (%) | 2258(43.72%) | 313(31.27%) | ＜.001 |  | 4202(41.53%) | 63(40.13%) | .72 |
| Single antiplatelet therapy, n (%) | 1010(19.55%) | 185(18.48%) | .43 |  | 1640(16.21%) | 27(17.2%) | .74 |
| Dual antiplatelet therapy, n (%) | 1097(21.24%) | 271(27.07%) | ＜.001 |  | 2271(22.45%) | 32(20.38%) | .54 |
| Oral anticoagulants, n (%) | 39(0.76%) | 27(2.7%) | ＜.001 |  | 18(0.18%) | 1(0.64%) | .69 |
| **Derivation cohort, Three Centers** | n=3872 | n=662 |  |  | n=8724 | n=141 |  |
| Proton pump Inhibitors, n (%) | 1405 (36.29%) | 189 (28.55%) | ＜.001 |  | 3258 (39.38%) | 52 (36.88%) | .91 |
| Single antiplatelet therapy, n (%) | 680 (17.56%) | 89 (13.44%) | .009 |  | 1258 (15.20%) | 23 (16.31%) | .53 |
| Dual antiplatelet therapy, n (%) | 771 (19.91%) | 184 (27.79%) | ＜.001 |  | 1893 (22.88%) | 27 (19.15%) | .47 |
| Oral anticoagulants, n (%) | 20 (0.52%) | 10 (1.51%) | .008 |  | 11 (0.13%) | 1 (0.71%) | .18 |
| **Validation cohort 1, Drum Tower** | n=1190 | n=164 |  |  | n=1376 | n=15 |  |
| Proton pump Inhibitors, n (%) | 827 (69.50%) | 98 (59.76%) | .01 |  | 942 (68.46%) | 11 (74.33%) | .79 |
| Single antiplatelet therapy, n (%) | 274 (23.03%) | 26 (15.85%) | .04 |  | 381 (27.69%) | 4 (26.67%) | 1 |
| Dual antiplatelet therapy, n (%) | 319 (26.81%) | 61 (37.20%) | .006 |  | 367 (26.67%) | 5 (33.33%) | .56 |
| Oral anticoagulants, n (%) | 18 (1.51%) | 10 (6.10%) | ＜.001 |  | 6 (0.44%) | 0 (0%) | 1 |
| **Validation cohort 2, MIMIC** | n=103 | n=175 |  |  | n=17 | n=1 |  |
| Proton pump Inhibitors, n (%) | 26 (25.24%) | 26 (14.85%) | .03 |  | 2 (11.76%) | 0 (0%) | 1 |
| Single antiplatelet therapy, n (%) | 56 (54.37%) | 70 (40%) | .02 |  | 11 (64.71%) | 0 (0%) | .39 |
| Dual antiplatelet therapy, n (%) | 7 (6.8%) | 26 (14.86%) | .02 |  | 1 (5.88%) | 0 (0%) | 1 |
| Oral anticoagulants, n (%) | 1 (0.97%) | 7 (4%) | .27 |  | 1 (5.88%) | 0 (0%) | 1 |

Abbreviations: GIB, gastrointestinal bleeding; MIMIC, Medical Information Mart for Intensive Care.

**Table S17. Univariate and Multivariate Analysis of Risk Factors for Gastrointestinal Bleeding after Coronary Artery Bypass Grafting in the High-Risk Subgroup of the Derivation Cohort**

| Variables | Univariate analysis | | Multivariate analysis | |
| --- | --- | --- | --- | --- |
|  | OR (95%CI) | *P* value | OR (95%CI) | *P* value |
| Demographics |  |  |  |  |
| Age (S.D.) | 1.063 (0.969-1.166) | .20 |  |  |
| Sex | 0.758 (0.689-0.835) | <.001 | 1.068 (0.952-1.198) | .26 |
| Body mass index (S.D.) | 0.874 (0.796-0.959) | .004 | 0.868 (0.783-0.962) | **.007** |
| Gastrointestinal bleeding history | 1.202 (0.559-2.584) | .64 |  |  |
| After percutaneous coronary intervention | 1.286 (1.016-1.627) | .04 | 1.34 (1.034-1.738) | **.03** |
| Chronic comorbidities |  |  |  |  |
| Anemia | 2.01 (1.68-2.405) | <.001 | 1.79 (1.449-2.212) | **<.001** |
| Coagulation disorders | 5.66 (4.047-7.914) | <.001 | 3.999 (2.678-5.971) | **<.001** |
| Hypertension | 0.969 (0.821-1.142) | .70 |  |  |
| Atrial fibrillation | 1.65 (1.264-2.154) | <.001 | 0.761 (0.548-1.056) | .10 |
| Diabetes | 0.973 (0.822-1.151) | .75 |  |  |
| Heart failure | 1.568 (1.321-1.861) | <.001 | 2.008 (1.647-2.449) | **<.001** |
| Cerebral vascular disease | 1.145 (0.948-1.383) | .16 |  |  |
| Peripheral vascular disease | 2.315 (1.653-3.241) | <.001 | 1.605 (1.081-2.383) | **.02** |
| Chronic kidney disease | 3.176 (2.609-3.867) | <.001 | 3.308 (2.572-4.254) | **<.001** |
| Gastrointestinal ulcer | 3.561 (2.48-5.114) | <.001 | 4.202 (2.759-6.399) | **<.001** |
| Gastritis | 1.346 (0.695-2.606) | .38 |  |  |
| Hyperlipidemia | 0.894 (0.757-1.056) | .19 |  |  |
| Valvular disease | 1.509 (1.267-1.797) | <.001 | 1.45 (1.185-1.774) | **<.001** |
| Admission examination (S.D.) |  |  |  |  |
| White blood cell count | 1.223 (1.115-1.341) | <.001 | 1.234 (1.11-1.372) | **<.001** |
| Red blood cell count | 0.836 (0.765-0.914) | <.001 | 0.982 (0.803-1.201) | .86 |
| Platelet count | 0.876 (0.803-0.955) | .003 | 0.878 (0.794-0.971) | **.01** |
| Hemoglobin | 0.82 (0.751-0.894) | <.001 | 0.902 (0.714-1.139) | .39 |
| Hematocrit | 0.836 (0.767-0.913) | <.001 | 1.045 (0.838-1.303) | .70 |
| Alanine aminotransferase | 1.136 (0.988-1.307) | .07 |  |  |
| Aspartate aminotransferase | 1.55 (1.351-1.777) | <.001 | 1.159 (0.97-1.386) | .10 |
| Bilirubin total | 1.302 (1.144-1.482) | <.001 | 1.251 (1.075-1.456) | **.004** |
| Albumin | 0.806 (0.734-0.885) | <.001 | 0.86 (0.774-0.955) | **.005** |
| Urea | 1.496 (1.322-1.693) | <.001 | 1.104 (0.934-1.304) | .25 |
| Creatinine | 1.598 (1.405-1.818) | <.001 | 1.049 (0.877-1.254) | .60 |
| Prothrombin time | 1.579 (1.386-1.798) | <.001 | 1.026 (0.741-1.421) | .88 |
| Activated partial thromboplastin time | 1.489 (1.303-1.701) | <.001 | 1.268 (1.09-1.474) | **.002** |
| International normalized ratio | 1.551 (1.379-1.744) | <.001 | 1.299 (0.968-1.744) | .081 |
| Lactate dehydrogenase | 1.624 (1.441-1.83) | <.001 | 1.274 (1.09-1.488) | **.002** |
| Ejection fraction drop value (per 5%) | 1.181 (1.077-1.294) | <.001 | 1.388 (1.251-1.54) | **<.001** |

| Variables | Univariate analysis | | Multivariate analysis | |
| --- | --- | --- | --- | --- |
|  | OR (95%CI) | *P* value | OR (95%CI) | *P* value |
| Preoperative medications |  |  |  |  |
| Proton pump Inhibitors | 0.702 (0.585-0.841) | <.001 | 0.477 (0.382-0.596) | **<.001** |
| Single antiplatelet therapy | 0.729 (0.575-0.925) | .009 | 0.906 (0.696-1.181) | .47 |
| Dual antiplatelet therapy | 1.548 (1.284-1.867) | <.001 | 1.937 (1.524-2.463) | **<.001** |
| Oral anticoagulants | 2.954 (1.376-6.339) | .005 | 2.521 (1.069-5.943) | **0.04** |

**Table S18. Univariate and Multivariate Analysis of Risk Factors for Gastrointestinal Bleeding after Coronary Artery Bypass Grafting in the High-Risk Subgroup of the Drum Tower Validation Cohort**

| Variables | | Univariate analysis | | | | Multivariate analysis | | | | |
| --- | --- | --- | --- | --- | --- | --- | --- | --- | --- | --- |
|  |  | OR (95%CI) | | *P* value | | OR (95%CI) | | *P* value | | |
| Demographics | |  | |  | |  | | |  | |
| Age (S.D.) | | 1.016 (0.851-1.214) | | .86 | |  | | |  | |
| Sex | | 0.651 (0.435-0.973) | | .04 | | 0.738 (0.45-1.21) | | | .23 | |
| Body mass index (S.D.) | | 1.084 (0.912-1.288) | | .36 | |  | | |  | |
| Gastrointestinal bleeding history | | 18.27 (7.395-45.139) | | <.001 | | 11.766 (3.675-37.674) | | | **<.001** | |
| After percutaneous coronary intervention | | 1.417 (0.893-2.25) | | .14 | |  | | |  | |
| Chronic comorbidities | |  | |  | |  | | |  | |
| Anemia | | 1.847 (1.262-2.703) | | .002 | | 1.951 (1.156-3.295) | | | **.01** | |
| Coagulation disorders | | 10.974 (4.117-29.251) | | <.001 | | 67.19 (19.64-229.87) | | | **<.001** | |
| Hypertension | | 1.621 (1.123-2.341) | | .01 | | 1.643 (1.043-2.588) | | | **.03** | |
| Atrial fibrillation | | 1.08 (0.679-1.717) | | .75 | |  | | |  | |
| Diabetes | | 1.302 (0.927-1.829) | | .13 | |  | | |  | |
| Heart failure | | 0.982 (0.706-1.364) | | .91 | |  | | |  | |
| Cerebral vascular disease | | 1.437 (0.994-2.075) | | .05 | |  | | |  | |
| Peripheral vascular disease | | 2.359 (1.472-3.782) | | <.001 | | 1.56 (0.86-2.829) | | | .14 | |
| Chronic kidney disease | | 3.023 (1.973-4.633) | | <.001 | | 2.712 (1.456-5.05) | | | **.002** | |
| Gastrointestinal ulcer | | 5.94 (2.829-12.472) | | <.001 | | 1.798 (0.558-5.79) | | | .33 | |
| Gastritis | | 0.779 (0.274-2.215) | | .64 | |  | | |  | |
| Hyperlipidemia | | 0.808 (0.581-1.124) | | .21 | |  | | |  | |
| Valvular disease | | 2.394 (1.699-3.375) | | <.001 | | 4.771 (3.017-7.547) | | | **<.001** | |
| Admission examination (S.D.) | |  | |  | |  | | |  | |
| White blood cell count | | 1.717 (1.42-2.077) | | <.001 | | 1.658 (1.284-2.141) | | | **<.001** | |
| Red blood cell count | | 0.729 (0.605-0.877) | | .001 | | 0.971 (0.634-1.485) | | | .89 | |
| Platelet count | | 0.688 (0.565-0.838) | | <.001 | | 0.645 (0.508-0.819) | | | **<.001** | |
| Hemoglobin | | 0.714 (0.59-0.863) | | <.001 | | 1.237 (0.707-2.162) | | | .46 | |
| Hematocrit | | 0.681 (0.566-0.819) | | <.001 | | 0.551 (0.317-0.959) | | | **.04** | |
| Alanine aminotransferase | | 1.229 (0.961-1.572) | | .1 | |  | | |  | |
| Aspartate aminotransferase | | 1.717 (1.356-2.174) | | <.001 | | 1.051 (0.746-1.48) | | | .78 | |
| Bilirubin total | | 1.324 (1.037-1.69) | | .02 | | 1.209 (0.888-1.647) | | | .23 | |
| Albumin | | 0.532 (0.409-0.692) | | <.001 | | 0.638 (0.463-0.879) | | | **.006** | |
| Urea | | 1.684 (1.323-2.143) | | <.001 | | 1.08 (0.761-1.534) | | | .67 | |
| Creatinine | | 1.838 (1.448-2.335) | | <.001 | | 1.053 (0.724-1.531) | | | .79 | |
| Prothrombin time | | 1.345 (1.035-1.747) | | .03 | | 0.777 (0.405-1.493) | | | .45 | |
| Activated partial thromboplastin time | | 1.241 (0.948-1.625) | | .12 | |  | | |  | |
| International normalized ratio | | 1.455 (1.145-1.85) | | .002 | | 1.42 (0.786-2.568) | | | .25 | |
| Lactate dehydrogenase | | 1.889 (1.536-2.324) | | <.001 | | 1.657 (1.235-2.221) | | | **.001** | |
| Ejection fraction drop value (per 5%) | | 1.328 (1.092-1.616) | | .005 | | 1.415 (1.112-1.799) | | | **.005** | |
| Variables | Univariate analysis | | | | Multivariate analysis | | | | |  |
|  | OR (95%CI) | | *P* value | | OR (95%CI) | | *P* value | | |  |
| Preoperative medications |  | |  | |  | |  | | |  |
| Proton pump Inhibitors | 0.652 (0.466-0.912) | | .01 | | 0.434 (0.273-0.69) | | **<.001** | | |  |
| Single antiplatelet therapy | 0.63 (0.406-0.978) | | .04 | | 1.469 (0.82-2.632) | | .20 | | |  |
| Dual antiplatelet therapy | 1.617 (1.149-2.276) | | .006 | | 1.823 (1.098-3.029) | | **.02** | | |  |
| Oral anticoagulants | 4.228 (1.917-9.326) | | <.001 | | 4.413 (1.506-12.931) | | **.007** | | |  |

**Table S19. Univariate and Multivariate Analysis of Risk Factors for Gastrointestinal Bleeding after Coronary Artery Bypass Grafting in the High-Risk Subgroup of the MIMIC Validation Cohort**

| Variables | Univariate analysis | | Multivariate analysis | |
| --- | --- | --- | --- | --- |
|  | OR (95%CI) | *P* value | OR (95%CI) | *P* value |
| Demographics |  |  |  |  |
| Age (S.D.) | 0.995 (0.77-1.287) | .97 |  |  |
| Sex | 0.796 (0.476-1.332) | .39 |  |  |
| Body mass index (S.D.) | 0.946 (0.735-1.217) | .67 |  |  |
| Gastrointestinal bleeding history | 1.348 (0.589-3.083) | .48 |  |  |
| After percutaneous coronary intervention | 0.711 (0.319-1.585) | .40 |  |  |
| Chronic comorbidities |  |  |  |  |
| Anemia | 1.414 (0.86-2.324) | .17 |  |  |
| Coagulation disorders | 5.112 (2.702-9.67) | <.001 | 3.781 (1.836-7.785) | **<.001** |
| Hypertension | 0.629 (0.385-1.027) | .06 |  |  |
| Atrial fibrillation | 3.001 (1.8-5.004) | <.001 | 2.575 (1.396-4.748) | **.002** |
| Diabetes | 0.805 (0.483-1.342) | .41 |  |  |
| Heart failure | 2.029 (1.237-3.328) | .005 | 1.239 (0.633-2.424) | .53 |
| Cerebral vascular disease | 1.408 (0.737-2.689) | .30 |  |  |
| Peripheral vascular disease | 1.352 (0.759-2.409) | .31 |  |  |
| Chronic kidney disease | 2.048 (1.223-3.428) | .006 | 1.514 (0.735-3.119) | .26 |
| Gastrointestinal ulcer | 6.182 (0.78-49.009) | .09 |  |  |
| Gastritis | 0.458 (0.12-1.748) | .25 |  |  |
| Hyperlipidemia | 0.697 (0.411-1.182) | .18 |  |  |
| Valvular disease | 1.419 (0.853-2.361) | .18 |  |  |
| Admission examination (S.D.) |  |  |  |  |
| White blood cell count | 1.447 (1.091-1.92) | .01 | 1.255 (0.895-1.761) | .19 |
| Red blood cell count | 0.857 (0.667-1.102) | .23 |  |  |
| Platelet count | 0.836 (0.628-1.114) | .22 |  |  |
| Hemoglobin | 0.755 (0.584-0.977) | .03 | 0.738 (0.539-1.009) | .06 |
| Hematocrit | 0.867 (0.672-1.12) | .28 |  |  |
| Alanine aminotransferase | 1.288 (0.811-2.045) | .28 |  |  |
| Aspartate aminotransferase | 1.48 (0.903-2.427) | .12 |  |  |
| Bilirubin total | 1.135 (0.707-1.824) | .60 |  |  |
| Albumin | 0.799 (0.616-1.035) | .09 |  |  |
| Urea | 1.405 (1.007-1.96) | .046 | 0.815 (0.48-1.385) | .45 |
| Creatinine | 1.947 (1.255-3.022) | .003 | 2.155 (1.075-4.32) | **.03** |
| Prothrombin time | 1.755 (1.175-2.622) | .006 | 1.381 (0.37-5.152) | .63 |
| Activated partial thromboplastin time | 1.532 (1.071-2.194) | .02 | 1.415 (0.914-2.191) | .12 |
| International normalized ratio | 1.75 (1.145-2.672) | .01 | 1.062 (0.274-4.123) | .93 |
| Lactate dehydrogenase | 1.474 (0.969-2.24) | .07 |  |  |
| Ejection fraction drop value (per 5%) | 2.2 (1.527-3.169) | <.001 | 2.28 (1.444-3.6) | **<.001** |

| Variables | Un ivariate analysis | | Multivariate analysis | |
| --- | --- | --- | --- | --- |
|  | OR (95%CI) | *P* value | OR (95%CI) | *P* value |
| Preoperative medications |  |  |  |  |
| Proton pump Inhibitors | 0.517 (0.281-0.95) | .03 | 0.507 (0.233-1.103 | .09 |
| Single antiplatelet therapy | 0.56 (0.342-0.915) | .02 | 0.684 (0.351-1.331 | .26 |
| Dual antiplatelet therapy | 2.821 (1.12-7.106) | .03 | 1.798 (0.554-5.834 | .33 |
| Oral anticoagulants | 4.25 (0.515-35.044) | .18 |  |  |
